# Utility of 5-(furan-2-yl)-3-(*p*-tolyl)-4,5-dihydro-1*H*-pyrazole-1-carbothioamide in the synthesis of heterocyclic compounds with antimicrobial activity

**DOI:** 10.1186/s13065-019-0566-y

**Published:** 2019-04-01

**Authors:** Abdou O. Abdelhamid, Ibrahim E. El Sayed, Yasser H. Zaki, Ahmed M. Hussein, Mangoud M. Mangoud, Mona A. Hosny

**Affiliations:** 1 Department of Chemistry, Faculty of Science, Cairo University, Giza, 12613 Egypt; 2 Department of Chemistry, Faculty of Science, El Menoufia University, Shebin El Koom, 32511 Egypt; 3 Department of Chemistry, Faculty of Science, Beni-Suef University, Beni-Suef, 62514 Egypt; 4 Environmental Research Department, National Center for Social and Criminological Research, IbnKhaldoun Square, Mohandesin, Zamalek, Giza, 11561 Egypt; 5 Department of Chemistry, Faculty of Women for Arts, Science and Education, Ain Shams University, Heliopolis, Cairo, 11757 Egypt

**Keywords:** Thiazoles, Hydrazonoyl halides, 1,3,4-Thiadiazoles, Urea derivatives, Pyrano[2,3-*d*]thiazoles, Antimicrobials

## Abstract

**Background:**

Pyrazolines show different biological activities. In recent years, interest in the chemistry of hydrazonoyl halides has been renewed. 1,3,4-Thiadiazoles are one of the most common heterocyclic pharmacophores with a wide range of biological activities.

**Results:**

Ethyl 2-(5-(furan-2-yl)-3-(*p*-tolyl)-4,5-dihydro-1*H*-pyrazol-1-yl)-4-methyl-thiazole-5-carboxylate, 2-(5-(furan-2-yl)-3-(*p*-tolyl)-4,5-dihydro-1*H*-pyrazol-1-yl)thiazol-4(5*H*)-one, and 1-(2-(5-(furan-2-yl)-3-(*p*-tolyl)-4,5-dihydro-1*H*-pyrazol-1-yl)-4-methylthiazol-5-yl)ethan-1-one were synthesized from the reaction of 5-(furan-2-yl)-3-(*p*-tolyl)-4,5-dihydro-1*H*-pyrazole-1-carbothioamide with different halogenated compounds. Thiazole, 1,3,4-thiadiazole and pyrano[2,3-*d*]thiazole derivatives were also synthesized. The structures of the newly synthesized compounds were elucidated based on elemental analysis, spectral data, and alternative synthetic routes whenever possible. Additionally, the newly synthesized compounds were screened for antimicrobial activity against various microorganisms.

**Conclusions:**

A new series of novel functionalized 1,3,4-thiadiazoles, 1,3-thiazoles, and pyrazoline-containing moieties were synthesized using hydrazonoyl halides as precursors and evaluated for their in vitro antibacterial, and antifungal activities. The antimicrobial results of the examined compounds revealed promising results and some derivatives have activities similar to the references used.
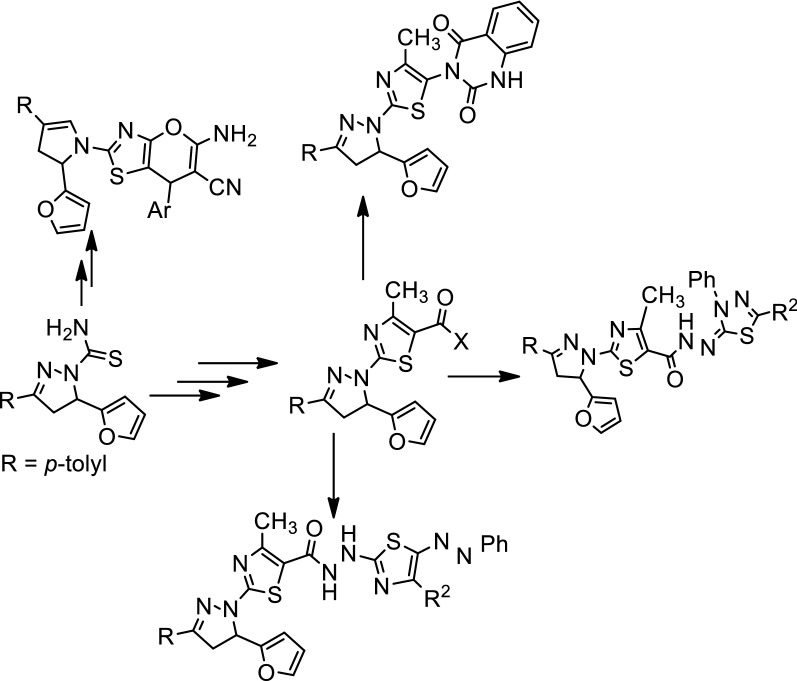

**Electronic supplementary material:**

The online version of this article (10.1186/s13065-019-0566-y) contains supplementary material, which is available to authorized users.

## Introduction

Pyrazolines show a variety of biological activities. They are antimicrobial [1–4], antifungal [5], anti-depressant [6], immunosuppressive [7], anticonvulsant [8–10], anti-tumor [11], anti-amoebic [12], antibacterial [13], anti-inflammatory [14], anticancer [15], and MAO inhibitory activity [16]. Hydrazonoyl halides have been widely used as reagents for the synthesis of various heterocyclic compounds [17, 18]. Thiazoles are used in drugs developed for the treatment of allergies [19], hypertension [20], inflammation [21], schizophrenia [22], bacterial infections [23], HIV [24], sleep disorders [25] and more recently, for the treatment of pain [26]. They are also used as fibrinogen receptor antagonists with antithrombotic activity [27], and as new inhibitors of bacterial DNA gyrase B [28]. Moreover, 1,3,4-thiadiazoles are among the most common heterocyclic pharmacophores. They display a broad spectrum of biological activities, including antimicrobial [29], anticancer [30, 31], antioxidant [32], anti-depressant [33], anticonvulsant [34, 35] and antihypertensive activities [36], as well as acetyl cholinesterase inhibition for the treatment of Alzheimer’s disease [37, 38]. In continuation of the author’s research work [39–45], the synthesis of some new thiazoles, 1,3,4-thiadiazoles and pyrano[2,3-*d*]thiazole from 5-(furan-2-yl)-3-(*p*-tolyl)-4,5-dihydro-1*H*-pyrazole-1-carbothioamide are reported herein.

## Results and discussion

**Scheme 1:**
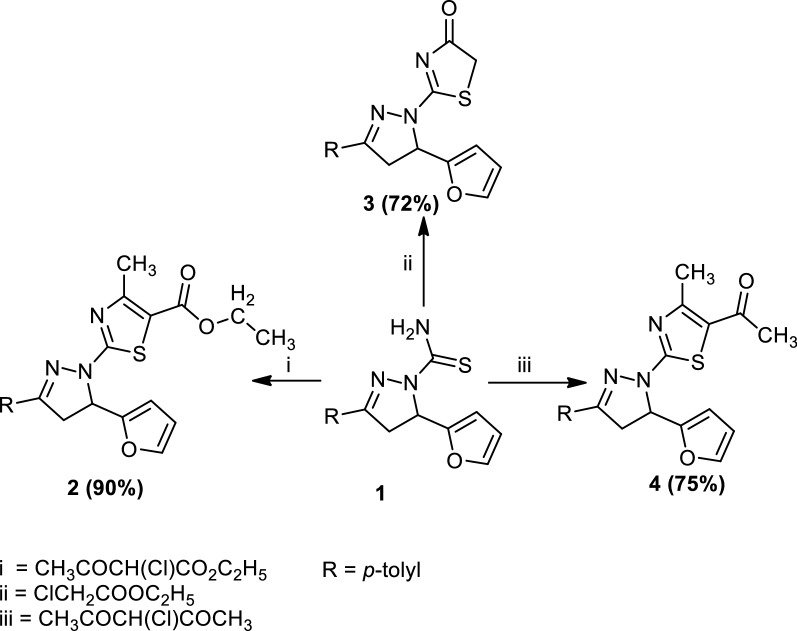
Synthesis of compounds (**2**–**4**)

The reaction of 5-(furan-2-yl)-3-(*p*-tolyl)-4,5-dihydro-1*H*-pyrazole-1-carbothioamide (**1**) with ethyl 2-chloro-3-oxobutanoate, ethyl 2-chloroacetate or 3-chloropentane-2,4-dione in ethanol containing an amount of triethylamine afforded ethyl 2-(5-(furan-2-yl)-3-(*p*-tolyl)-4,5-dihydro-1*H*-pyrazol-1-yl)-4-methylthiazole-5-carboxylate (**2**), 2-(5-(furan-2-yl)-3-(*p*-tolyl)-4,5-dihydro-1*H*-pyrazol-1-yl)thiazol-4(5*H*)-one (**3**) and 1-(2-(5-(furan-2-yl)-3-(*p*-tolyl)-4,5-dihydro-1*H*-pyrazol-1-yl)-4-methylthiazol-5-yl)ethan-1-one (**4**), respectively (Scheme 1).

**Scheme 2:**
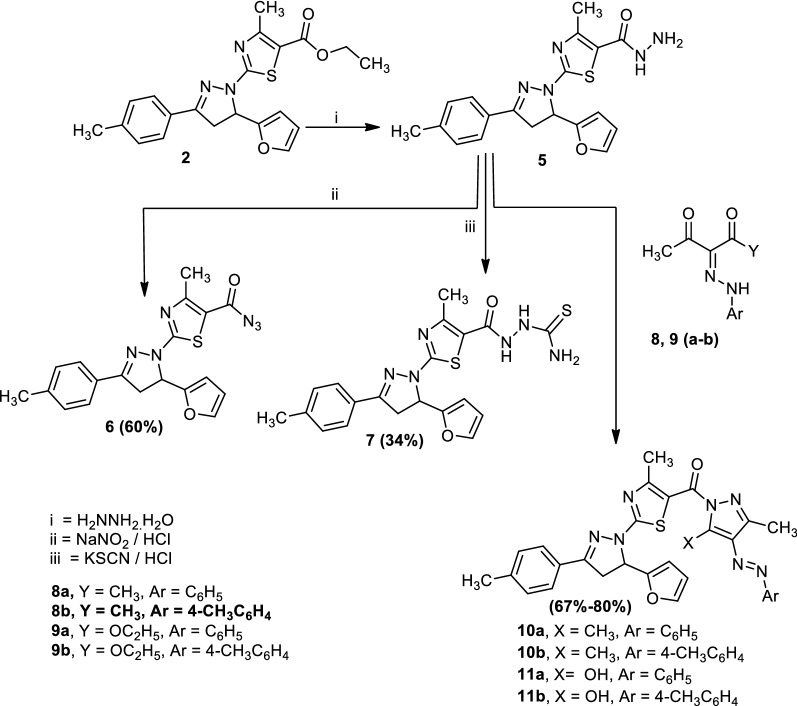
Synthesis of compounds (**6**, **7**, **10a**, **10b**, **11a** and **11b**)

The structures of the compounds (**2**–**4**) were clarified by elemental analyses, FTIR, MS, NMR spectra and chemical transformation. Compound (**2**) reacted with hydrazine hydrate to afford 2-(5-(furan-2-yl)-3-(*p*-tolyl)-4,5-dihydro-1*H*-pyrazol-1-yl)-4-methylthiazole-5-carbohydrazide (**5**) (Scheme 2). The structure of compound (**5**) was elucidated by elemental analyses, spectral data, and chemical transformations. Compound (**5**) reacted with nitrous acid, potassium thiocyanate, 3-(2-arylhydrazono)pentane-2,4-dione (**8a** and **8b**) or ethyl 2-(2-arylhydrazono)-3-oxobutanoate (**9a** and **9b**) to afford the following: 2-(5-(furan-2-yl)-3-(*p*-tolyl)-4,5-dihydro-1*H*-pyrazol-1-yl)-4-methylthiazole-5-carbonyl azide (**6**), 2-(2-(5-(furan-2-yl)-3-(*p*-tolyl)-4,5-dihydro-1*H*-pyrazol-1-yl)-4-methylthiazole-5-carbonyl)hydrazine-1-carbothioamide (**7**), (3,5-dimethyl-4-(phenyldiazenyl)-1*H*-pyrazol-1-yl)(2-(5-(furan-2-yl)-3-(*p*-tolyl)-4,5-dihydro-1*H*-pyrazol-1-yl)-4-methylthiazol-5-yl)methanone (**10a**), (3,5-dimethyl-4-(*p*-tolyldiazenyl)-1*H*-pyrazol-1-yl)(2-(5-(furan-2-yl)-3-(*p*-tolyl)-4,5-dihydro-1*H*-pyrazol-1-yl)-4-methylthiazol-5-yl)methanone (**10b**), 2-(2-(5-(furan-2-yl)-3-(*p*-tolyl)-4,5-dihydro-1*H*-pyrazol-1-yl)-4-methylthiazole-5-carbonyl)-5-methyl-4-(2-phenylhydrazono)-2,4-dihydro-3*H*-pyrazol-3-one (**11a**) and 2-(2-(5-(furan-2-yl)-3-(*p*-tolyl)-4,5-dihydro-1*H*-pyrazol-1-yl)-4-methylthiazole-5-carbonyl)-5-methyl-4-(2-(*p*-tolyl)hydrazono)-2,4-dihydro-3*H*-pyrazol-3-one (**11b**), respectively (Scheme 2). The structures of compounds (**6**, **7**, **10a** and **10b**) and (**11a** and **11b**) were confirmed by elemental analyses, spectral data and chemical transformations whenever possible.

**Scheme 3:**
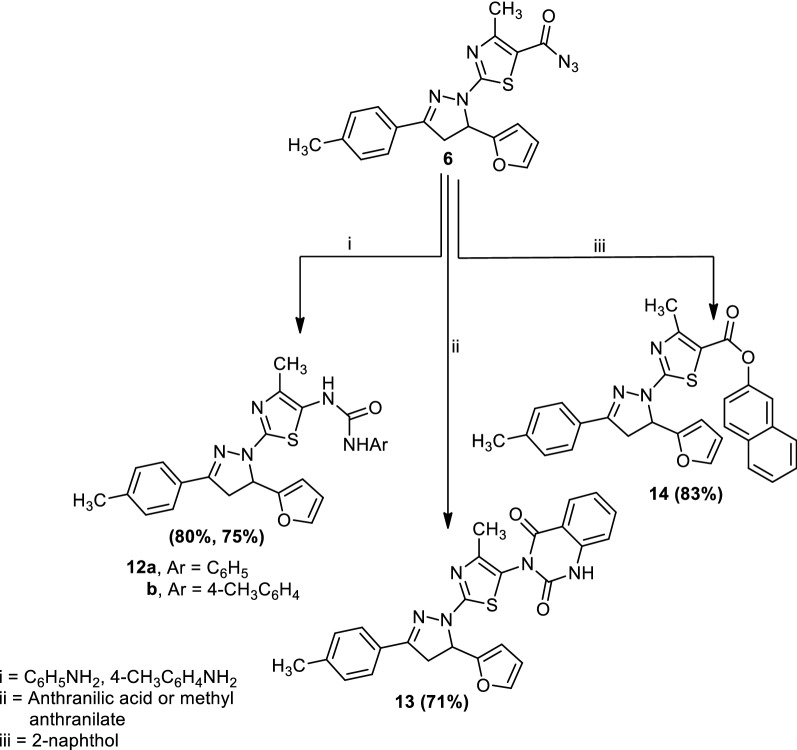
Synthesis of compounds (**12**–**14**)

Treatment of 2-(5-(furan-2-yl)-3-(*p*-tolyl)-4,5-dihydro-1*H*-pyrazol-1-yl)-4-methylthiazole-5-carbonyl azide (**6**) with aniline, 4-toluidine or anthranilic acid in boiling dioxane gave1-(2-(5-(furan-2-yl)-3-(*p*-tolyl)-4,5-dihydro-1*H*-pyrazol-1-yl)-4-methylthiazol-5-yl)-3-phenylurea (**12a**), 1-(2-(5-(furan-2-yl)-3-(*p*-tolyl)-4,5-dihydro-1*H*-pyrazol-1-yl)-4-methylthiazol-5-yl)-3-(*p*-tolyl)urea (**12b**) and 3-(2-(5-(furan-2-yl)-3-(*p*-tolyl)-4,5-dihydro-1*H*-pyrazol-1-yl)-4-methylthiazol-5-yl)quinazoline-2,4(1*H*,3*H*)-dione (**13**), respectively. Also, compound (**6**) reacted with 2-naphthol in boiling benzene to afford naphthalen-2-yl(2-(5-(furan-2-yl)-3-(*p*-tolyl)-4,5-dihydro-1*H*-pyrazol-1-yl)-4-methylthiazole-5-carboxylate (**14**) (Scheme 3). The structures of compounds (**12**–**14**) were confirmed by elemental analyses, spectral data and an alternative synthetic route. Thus, compound (**6**) reacted with methyl anthranilate in dioxane to afford a product identical in all aspects (mp, mixed mp and spectra) to compound (**13**).

**Scheme 4:**
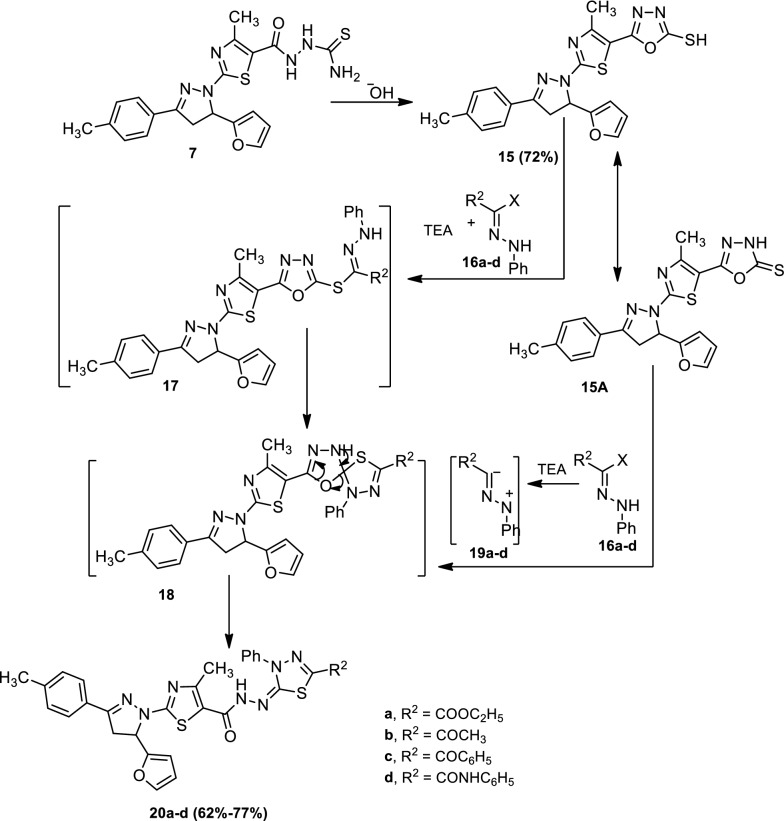
Synthesis of compounds (**15**) and (**20a**–**d**)

Next, treatment of 2-(2-(5-(furan-2-yl)-3-(*p*-tolyl)-4,5-dihydro-1*H*-pyrazol-1-yl)-4-methylthiazole-5-carbonyl)hydrazine-1-carbothioamide (**7**) with sodium hydroxide yielded 5-(2-(5-(furan-2-yl)-3-(*p*-tolyl)-4,5-dihydro-1*H*-pyrazol-1-yl)-4-methylthiazol-5-yl)-1,3,4-oxadiazole-2-thiol (**15**). The latter reacted with the appropriate hydrazonoyl halides (**16a**–**d**) in refluxing chloroform in the presence of triethylamine to give *N’*-(5-substituted-3-phenyl-1,3,4-thiadiazol-2(3*H*)-ylidene)-2-(5-(furan-2-yl)-3-(*p*-tolyl)-4,5-dihydro-1*H*-pyrazol-1-yl)-4-methylthiazole-5-carbohydrazide (**20a**–**d**). The mechanism outlined in Scheme 4 seemed to be the most plausible pathway for the formation of (**20**) from the reaction of (**15**) or (**15a**) with (**16**) by two possible pathways. The first pathway was via 1,3-addition of the thiol tautomer (**15**) to the nitrilimine (**19a**–**d**) (which produced in situ from the reaction of hydrazonoyl halide [16a–d] with triethylamine) to give the thiohydrazonate ester (**17**) that underwent nucleophilic cyclization to yield *spiro* compound (**18**). The latter underwent ring opening and cyclization to yield (**20**). The second pathway was via 1,3-cycloaddition of nitrilimine (**19**) to the C=S double bond of (**15a**) to give (**18**) directly (Scheme 4). Attempts to isolate the thiohydrazonate ester (**17**) or the intermediate (**18**) did not succeed, even under mild conditions, as these two compounds readily underwent in situ cyclization to give the final isolable product (**20**), as shown in Scheme 4.

**Scheme 5:**
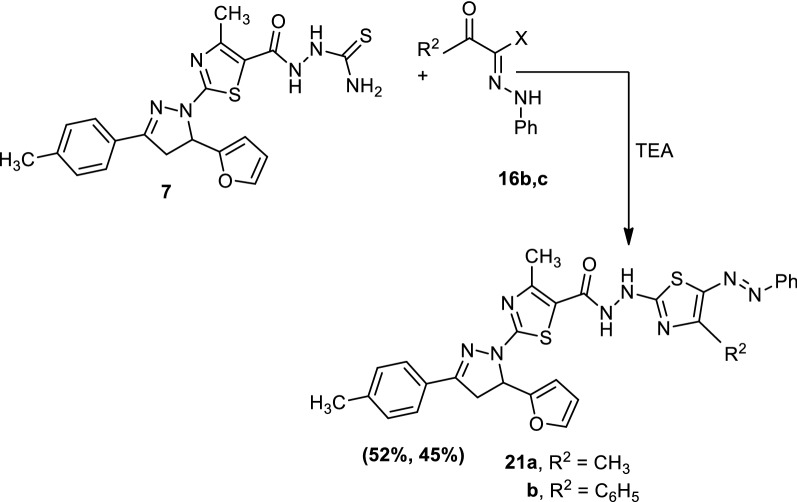
Synthesis of compounds (**21a** and **21b**)

Treatment of 2-(2-(5-(furan-2-yl)-3-(*p*-tolyl)-4,5-dihydro-1*H*-pyrazol-1-yl)-4-methylthiazole-5-carbonyl)hydrazine-1-carbothioamide (**7**) with the appropriate hydrazonoyl halides (**16b**) and (**16c**) in ethanolic triethylamine afforded 2-(5-(furan-2-yl)-3-(*p*-tolyl)-4,5-dihydro-1*H*-pyrazol-1-yl)-4-methyl-*N’*-(4-methyl-5-(phenyldiazenyl)-thiazol-2-yl)thiazole-5-carbohydrazide (**21a**) and 2-(5-(furan-2-yl)-3-(*p*-tolyl)-4,5-dihydro-1*H*-pyrazol-1-yl)-4-methyl-*N’*-(4-phenyl-5-(phenyldiazenyl)thiazol-2-yl)thiazole-5-carbohydrazide (**21b**), respectively (Scheme 5). The structures of compounds (**21a** and **21b**) were confirmed by elemental analyses and spectral data.

**Scheme 6:**
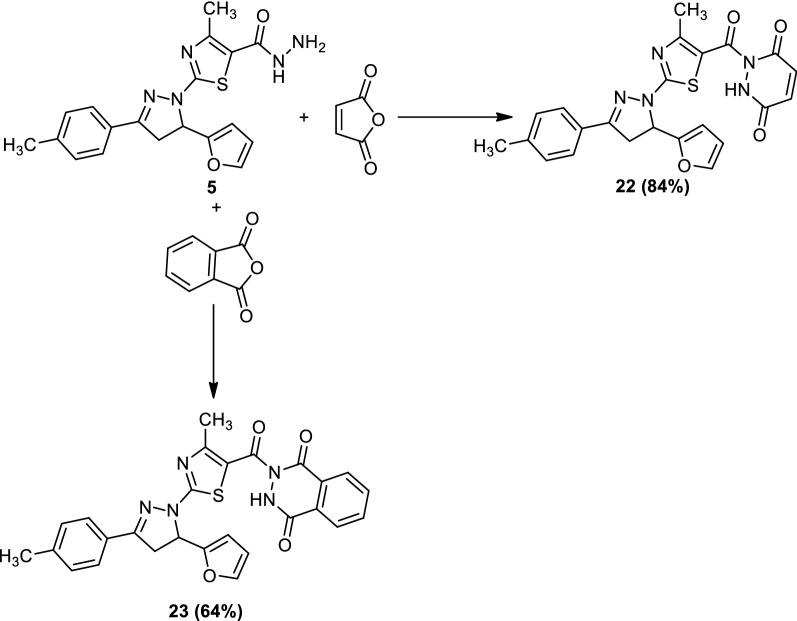
Synthesis of compounds (**22**) and (**23**)

On the other hand, the treatment of compound (**5**) with maleic anhydride and phthalic anhydride afforded 1-(2-(5-(furan-2-yl)-3-(*p*-tolyl)-4,5-dihydro-1*H*-pyrazol-1-yl)-4-methylthiazole-5-carbonyl)-1,2-dihydropyridazine-3,6-dione (**22**) and 2-(2-(5-(furan-2-yl)-3-(*p*-tolyl)-4,5-dihydro-1*H*-pyrazol-1-yl)-4-methylthiazole-5-carbonyl)-2,3-dihydro-phthalazine-1,4-dione (**23**), respectively (Scheme 6). The structures of compounds (**22**) and (**23**) were elucidated by elemental analyses and spectral data (*cf*. Experimental).

**Scheme 7:**
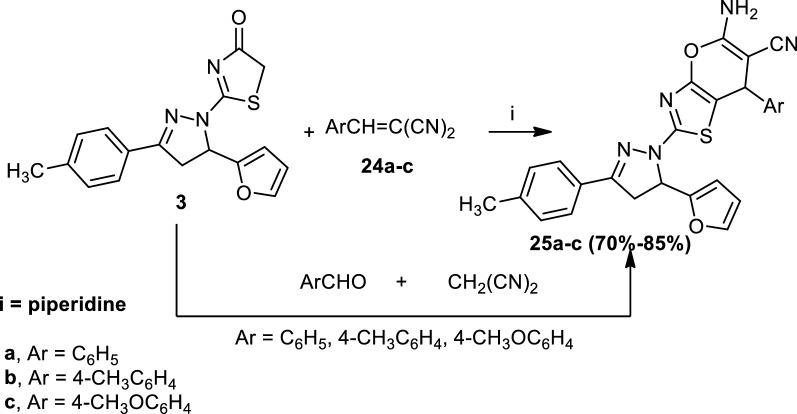
Synthesis of compounds (**25a**–**c**)

Finally, treatment of 2-(5-(furan-2-yl)-3-(*p*-tolyl)-4,5-dihydro-1*H*-pyrazol-1-yl)thiazol-4(5*H*)-one (**3**) with arylidenemalononitriles (**24a**–**c**) in boiling ethanol containing a catalytic amount of piperidine afforded 5-amino-2-(5-(furan-2-yl)-3-(*p*-tolyl)-4,5-dihydro-1*H*-pyrazol-1-yl)-7-aryl-7*H*-pyrano[2,3-*d*]thiazole-6-carbonitrile (**25a**–**c**). The structures of compounds (**25a**–**c**) were elucidated by elemental analyses, spectral data and a synthetic route. Thus, the infrared (IR) spectrum of compound (**25a**) showed bands at 3388 and 3175 cm^−1^, which corresponded to the NH_2_ group. Furthermore, a mixture of malononitrile, an appropriate aldehyde and 2-(5-(furan-2-yl)-3-(*p*-tolyl)-4,5-dihydro-1*H*-pyrazol-1-yl)thiazol-4(5*H*)-one (**3**) in ethanol containing a few drops of piperidine as a catalyst was heated under reflux to afford products identical in all aspects (mp, mixed mp and spectra) with (**25a**–**c**), respectively (Scheme 7).

## Antimicrobial activity

For their in vitro antibacterial activity against *Streptococcus pneumonia* and *Bacillus subtilis* and *Pseudomonas aeruginosa* and *Escherichia coli*, twenty-one of the newly synthesized target compounds were assessed. They were also assessed against a representative panel of fungal strains for their in vitro antifungal activity (i.e., *Aspergillus fumigatus* and *Candida albicans*). Ampicillin and gentamicin for in vitro antibacterial activity were used as reference drugs; While Amphotericin B was used for in vitro antifungal activity as a reference drug. Examinations were conducted at Al-Azhar University’s Regional Center for Mycology and Biotechnology (Nasr City, Cairo, Egypt). Microbes were obtained from the Microbiological Resource Center, Faculty of Agriculture, Ain Shams University, Cairo, Egypt.

Table 1 summarizes the test results for antimicrobial effects

**Table 1:** Mean zone of inhibition beyond well diameter (6 mm) produced on a range of clinically pathogenic microorganisms using a 5 mg/mL concentration of tested samples

Compound no.	Microorganisms					
	Fungi		Gram-Positive Bacteria		Gram-Negative Bacteria	
	*AF*	*CA*	*SP*	*BS*	*PA*	*EC*
**3**	16.2	12.5	16.8	14.6	12.1	12.8
**4**	15.7	13.2	10.5	13.6	12.6	11.2
**10a**	12.6	11.2	0	0	0	0
**10b**	17.3	16.9	17.3	18.3	18.3	22.6
**11a**	21.2	19.6	23.8	23.8	17.3	19.9
**11b**	15.7	13.3	0	0	0	0
**12a**	18.9	15.4	15.7	14.1	10.8	11.1
**12b**	16.7	18.1	16.7	21.1	10.7	9.9
**13**	19.1	16.9	13.6	14.7	12.1	10.4
**14**	15.7	14.1	17.2	14.9	15.2	17.2
**15**	16.4	12.7	19.9	18.4	11.6	10.9
**20a**	20.8	16.8	13.1	10.8	13.4	12.3
**20b**	26.8	15.3	11.2	12.7	9.8	11.3
**20c**	15.9	17.1	18.7	15.4	11.7	10.3
**20d**	20.6	15.8	18.9	12.7	11.3	9.9
**21a**	17.7	18.2	19.2	15.4	10.2	8.8
**21b**	19.1	18.9	17.3	17.7	0	9.9
**22**	23.8	32.4	13.2	13.3	0	10.2
**23**	18.8	15.6	17.9	13.3	11.4	10.7
**25a**	18.4	16.3	12.6	13.2	10.1	10.9
**25b**	0	0	12.7	14	9.7	8.3
**Amphotericin B**	23.7	25.4	–	–	–	–
**Ampicillin**	–	–	23.8	32.4	–	–
**Gentamicin**	–	–	–	–	17.3	19.9

- *Streptococcus pneumonia, Bacillus subtilis, Pseudomonas aeruginosa* and *Escherichia coli* were resistant to compounds (**10a** and **11b**).
- *Aspergillus fumigatus* was susceptible to compounds (**11a**), (**20a**), (**20b**), (**20d**), and (**22**).
- *Aspergillas fumigates* and *Candida albicans* were resistant to compound (**25b**).
- *Candida albicans* was moderate of all compounds in the table compared to amphotericin B.
- *Streptococcus pneumonia, Pseudomonas aeruginosa* and *Escherichia coli* were moderate of all compounds in the table compared to ampicillin and gentamicin.

According to these results, we can suggest the following structure activity relationships:

A. In the thiazoles (**3**), (**4**), and (**14**)
  1. Attachment of C_10_H_7_OCO group in (**14**) at position 5 in the thiazole ring is very important for antimicrobial activity and increases the activity towards Gram-negative bact.
  2. Attachment of H or CH_3_CO group at position 5 in the thiazole ring showed a moderate antimicrobial activity for all microorganisms in Table 1.
B. In the thiazolyluera (**12a**) and (**12b**)
  1. Attachment of PhNHCONH or 4-CH_3_C_6_H_4_NHCONH group in (**12a**) or (**12b**) at position 5 in the thiazole ring showed a moderate antimicrobial activity for all microorganisms in Table 1.
C. In the thiazolylpyrazoles **10**, **11**(**a**–**b**)
  1. Attachment of methyl and –N=NPh groups in (**10a**) and attachment of OH and –N=NPh groups in (**11b**) at positions 3, 4 respectively, in the moiety of the pyrazole ring had no activity against all the tested Gram-positive and Gram-negative bact. but had moderate activity against test fungi.
  2. Attachment of OH and –N=NPh groups in (**11a**) at position 3 and position 4 in the moiety of the pyrazole ring displayed potent effect against all the tested Gram-positive, Gram-negative bact. and fungi.
  3. Attachment of CH_3_ and 4–CH_3_C_6_H_4_N=N groups in (**10b**) at position 3 and position 4 in the moiety of the pyrazole ring displayed potent effect against Gram-negative bact., a moderate activity against Gram-positive bact. and fungi.
D. In the thiazolylquinazolinedione (**13**)
  1. Attachment of quinazoline-2,4(1*H*,3*H*)-dione ring at position 5 in the thiazole ring showed a moderate antimicrobial activity for all microorganisms in Table 1.
E. In the thiazolyloxadiazole (**15**)
  1. Attachment of 1,3,4-oxadiazole-2-thiole ring at position 5 in the thiazole ring showed a moderate antimicrobial activity for all microorganisms in Table 1.
F. In the thiazolylthiadiazole carbohydrazide (**20a**–**d**)
  1. Attachment of C_2_H_5_CO_2_ group in (**20a**) at position 2 in the moiety of the 1,3,4-thiadiazole ring displayed potent effect against *Af* fungus, moderate activity against Gram-positive bact., Gram-negative bact., and *CA* fungus.
  2. Attachment of CH_3_CO group in (**20b**) at position 5 in the moiety of the 1,3,4-thiadiazole ring displayed potent effect against *Af* fungus, moderate activity against Gram-positive bact., Gram-negative bact., and *CA* fungus.
  3. Attachment of C_6_H_5_CO group in (**20c**) at position 5 in the moiety of the 1,3,4-thiadiazole ring displayed a moderate antimicrobial activity for all microorganisms in Table 1.
  4. Attachment of C_6_H_5_CONH group in (**20d**) at position 2 in the moiety of the 1,3,4-thiadiazole ring displayed potent effect against *Af* fungus, moderate activity against Gram-positive bact., Gram-negative bact., and *CA* fungus.
G. In the thiazolylthiazole carbohydrazide (**21a**, **b**)
  1. Attachment of CH_3_– group in (**21a**) at position 4 in the moiety of the thiazole ring displayed a moderate antimicrobial activity for all microorganisms in Table 1.
  2. Attachment of C_6_H_5_– group in (**21b**) at position 4 in the moiety of the thiazole ring displayed a moderate antimicrobial activity for all microorganisms in Table 1 except *PA* which has no activity.
H. In the thiazolylpyridazine-3,6-dione (**22**)
  Attachment of carbonyl-1,2-dihydropyridazine-3,6-dione group at position 5 in the thiazole ring displayed potent effect against fungi and a moderate activity against Gram-positive bact., and Gram-negative bact. except *PA* which has no activity.
I. In the thiazolylphthalazine-1,4-dione (**23**)
  Attachment of carbonyl-2,3-dihydrophthalazine-1,4-dione group at position 5 in the thiazole ring showed a moderate antimicrobial activity for all microorganisms in Table 1.
J. In the thiazolylpyrano[2,3-*d*]thiazole-6-carbonitrile (**25a**, **b**)
  1. Attachment of C_6_H_5_- group in (**25a**) at position 7 in the moiety of the pyrano[2,3-*d*]thiazole-6-carbonitrile ring displayed a moderate antimicrobial activity for all microorganisms in Table 1.
  2. Attachment of 4–CH_3_C_6_H_4_ group in (**25b**) at position 7 in the moiety of the pyrano[2,3-*d*]thiazole-6-carbonitrile ring displayed a moderate activity against Gram-positive bact., and Gram-negative bact. and has no activity on fungi.

## Conclusions

Hydrazonoyl halides were used as precursors to synthesize a new series of novel functionalized 1,3,4-thiadiazoles, 1,3-thiazoles and pyrazoline-containing moieties. Antibacterial and antifungal activities of these compounds were assessed in vitro. *Streptococcus pneumonia*, *Bacillus subtilis*, *Pseudomonas aeruginosa* and *Escherichia coli* were resistant to compounds (**10a**), (**11b**) on the basis of the screening results. *Aspergillus fumigatus* was susceptible to compounds (**11a**), (**20a**), (**20b**), (**20d**), and (**22**). *Candida albicans* compared to amphotericin B was moderate for all compounds. Compared to ampicillin and gentamycin*, Streptococcus pneumonia, Pseudomonas aeruginosa* and *Escherichia coli* were moderate for all compounds.

## Experimental

### General information

An electrothermal device (Bibby Sci. Lim. Stone, Staffordshire, UK) has been used to determine all melting points and they are uncorrected. A FT—IR 8201 PC spectrophotometer (Shimadzu, Tokyo, Japan) was used to determine the IR spectra. On Varian Mercury VX-300 NMR spectrometer (Varian, Inc., Karlsruhe, Germany) operating at 300 MHz (^1^H NMR), the ^1^H-NMR spectra were recorded in CDCl_3_ and DMSO-*d*_6_ solutions. The chemical shifts are expressed in δ ppm units using TMS as an internal reference. On a Shimadzu GC–MS QP1000 EX instrument (Tokyo, Japan) mass spectra were recorded. Elemental analyses were performed at the University of Cairo’s Microanalytical Center. As previously reported, hydrazonoyl halides [46–49] and 5-(furan-2-yl)-3-(*p*-tolyl)-4,5-dihydro-1*H*-pyrazole-1-carbothioamide [39] Additional file 1: Figure S1 were prepared. In the Regional Center for Mycology and Biotechnology, Al-Azhar University, Cairo, Egypt, antimicrobial screening was conducted.

### Compounds (**2**–**4**)

#### General procedure

A mixture of compound (**1**) (2.85 g, 5 mmol), and the appropriate halogenated reagents (ethyl 2-chloro-3-oxobutanoate, ethyl 2-chloroacetate, or 3-chloropentane-2,4-dione) (10 mmol) in ethanol (20 mL) containing a catalytic amount of triethylamine was refluxed for 2 h. The reaction mixture was left to cool to room temperature. The formed solid was filtered off, dried, and recrystallized from an appropriate solvent to obtain the corresponding compounds (**2**–**4**), respectively.

##### Compound (**2**). Additional file 2: Figure S2

Yellow solid from ethanol, yield (3.56 g, 90%), mp: 124–125 °C; IR (KBr, cm^−1^): 3115 (=C–H aromatic), 3068 (=C–H), 2976 (–C–H), 1697 (C=O); ^1^H NMR: δ: 1.23 (t, 3H, *J* = 7.5 Hz, –OCH_2_CH_3_), 2.36 (s, 3H, 4–CH_3_-thiazole), 2.50 (s, 3H, 4–CH_3_C_6_H_4_), 3.40 (dd, 1H, *J *= 13.6 Hz, 16.2 Hz, pyrazoline-H), 3.80 (dd, 1H, *J *= 13.6 Hz, 16.2 Hz, pyrazoline-H), 4.15 (q, 2H, *J* = 7.5 Hz, –OCH_2_CH_3_), 5.56 (dd, 1H, *J *= 13.6 Hz, 16.2 Hz, pyrazoline-H), 6.40–7.72 (m, 7H, ArH’s + furyl-H’s); ^13^C-NMR (DMSO-*d*6) δ:14.2 (CH_3_), 17.0 (CH_3_), 21.4 (CH_3_), 35.7 (CH_2_), 60.3 (OCH_2_), 61.8 (CH), 94.5, 110.6, 117.0, 125.7, 129.2, 130.0, 140.7, 149.8, 150.9, 152.5, 163.6. MS (*m/z*): 396 (M+ 1, 2), 395 (M+, 10), 347 (6), 255 (10), 228 (28), 169 (100), 168 (66), 167 (40), 84 (12), 77 (38), 30 (26); *Anal.*Calcd. for C_21_H_21_N_3_O_3_S (395.47): C, 63.78; H, 5.35; N, 10.63; S, 8.11; found: C, 63.75; H, 5.36; N, 10.65; S, 8.11.

##### Compound (**3**). Additional file 3: Figure S3

Pale yellow solid from dioxane, yield (2.34 g, 72%), mp: 244–245 °C; IR (KBr, cm^−1^): 3143 (=C–H aromatic), 3039 (=C–H), 2991 (–C–H), 1697 (C=O); ^1^H NMR: δ: 2.44 (s, 3H, 4-CH_3_C_6_H_4_), 3.61 (dd, 1H, *J *= 13.6 Hz, 16.2 Hz, pyrazoline-H), 3.92 (s, 2H, thiazole-H), 3.95 (dd, 1H, *J *= 13.6 Hz, 16.2 Hz, pyrazoline-H), 5.85 (q, 1H, *J *= 13.6 Hz, 16.2 Hz, pyrazoline-H), 6.42–7.76 (m, 7H, ArH’s + furyl-H’s); ^13^C-NMR (DMSO-*d*6) δ: 21.4 (CH_3_), 35.8 (CH_3_),38.8 (CH_2_), 61.8 (CH), 94.4, 106.5, 125.7, 129.2, 130.0, 140.7, 142.1, 150.8, 154.1, 173.5, 187.6. MS (*m/z*): 327 (M+ 2, 1), 326 (M+ 1, 10), 325 (M+, 50), 308 (47), 293 (100), 275 (51), 101 (35), 77 (41), 69 (68), 59 (48), 44 (36), 30 (41); *Anal.* Calcd. for C_17_H_15_N_3_O_2_S (325.38): C, 62.75; H, 4.65; N, 12.91; S, 9.85; found: C, 62.71; H, 4.67; N, 12.92; S, 9.86.

##### Compound (**4**). Additional file 4: Figure S4

Yellow solid from glacial acetic acid, yield (2.74 g, 75%), mp: 176–177 °C; IR (KBr, cm^−1^): 3134 (=C–H aromatic), 3026 (=C–H), 2966 (–C–H), 1751 (C=O); ^1^H NMR: δ:2.36 (s, 3H, 4-CH_3_C_6_H_4_), 2.46 (s, 3H, 4-CH_3_-thiazole), 2.50 (s, 3H, CO–CH_3_), 3.50 (dd, 1H, *J *= 13.6 Hz, 16.2 Hz, pyrazoline-H), 3.85 (dd, 1H, *J *= 13.6 Hz, 16.2 Hz, pyrazoline-H), 5.79 (dd, 1H, *J *= 13.6 Hz, 16.2 Hz, pyrazoline-H), 6.40 (m, 2H, furyl-H), 7.29–7.72 (m, 5H, ArH’s + 1furyl-H); ^13^C-NMR (DMSO-*d*6) δ:17.0 (CH_3_), 21.3 (CH_3_), 28.6 (CH_3_), 35.7 (CH_2_), 61.7 (CH), 94.5, 110.6, 113.5, 125.8, 129.2, 130.0, 140.8, 142.2, 149.9, 151.1, 153.5, 153.9, 191.2. MS (*m/z*): 367 (M+ 2, 2), 366 (M+ 1, 9), 365 (M+, 38), 264 (16), 263 (14), 224 (10), 223 (11), 205 (8), 142 (25), 114 (100), 44 (16); *Anal.* Calcd. for C_20_H_19_N_3_O_2_S (365.45): C, 65.73; H, 5.24; N, 11.50; S, 8.77; found: C, 65.71; H, 5.25; N, 11.50; S, 8.76.

##### Compound (**5**). Additional file 5: Figure S5

A mixture of ethyl 2-(5-(furan-2-yl)-3-(*p*-tolyl)-4,5-dihydro-1*H*-pyrazol-1-yl)-4-methylthiazole-5-carboxylate (**2**) (3.95 g, 10 mmol), and hydrazine hydrate (20 mL) was heated under reflux for 12 h. The reaction mixture was left to cool to room temperature. The formed precipitate was filtered off, washed with ethanol, and recrystallized from glacial acetic acid to obtain compound (**5**) as a white solid yield (1.52 g, 40%), mp: 204–207 °C; IR (KBr, cm^−1^): 3400 (N–H), 3028 (=C–H), 2924 (–C–H), 1590 (C=O); ^1^H NMR: δ: 2.31 (s, 3H, 4-CH_3_C_6_H_4_), 2.36 (s, 3H, 4-CH_3_-thiazole), 3.47 (dd, 1H, *J *= 13.6 Hz, 16.2 Hz, pyrazoline-H), 3.64 (dd, 1H, *J *= 13.6 Hz, 16.2 Hz, pyrazoline-H), 3.71 (s, 1H, N–H), 5.59 (dd, 1H, *J *= 13.6 Hz, 16.2 Hz, pyrazoline-H), 6.29–7.64 (m, 9H, ArH’s + 2N–H + furyl-H’s); ^13^C-NMR (DMSO-*d*6) δ:17.0 (CH_3_), 21.4 (CH_3_), 35.7 (CH_2_), 61.8 (CH), 94.5, 107.8, 110.6, 1253.7, 129.2, 130.0, 140.7, 142.2, 145.5, 149.9, 151.1, 154.0, 185.8. MS (*m/z*): 383 (M+ 2, 3), 382 (M+ 1, 22), 381 (M+, 100), 200 (54), 183 (13), 115 (14), 152 (22), 104 (19), 103 (40), 91 (19), 43 (87); *Anal.* Calcd. for C_19_H_19_N_5_O_2_S (381.45): C, 59.82; H, 5.02; N, 18.36; S, 8.41; found: C, 59.79; H, 5.03; N, 18.37; S, 8.42.

##### Compound (**6**). Additional file 6: Figure S6

Sodium nitrite (0.69 g, 10 mmol) was dissolved in the least amount of water, and then added dropwise, to a suspension of 2-(5-(furan-2-yl)-3-(*p*-tolyl)-4,5-dihydro-1*H*-pyrazol-1-yl)-4-methylthiazole-5-carbohydrazide (**5**) (3.8 g, 10 mmol) in 37% HCl (10 mmol) at 0–5 °C. The formed precipitate was filtered off, washed with water, and recrystallized from ethanol to obtain compound (**6**) as a brownish yellow solid, yield (2.35 g, 60%), mp: 138–140 °C; IR (KBr, cm^−1^): 3032 (=C–H), 2921 (–C–H), 2126 (-N_3_), 1664 (C=O); ^1^H NMR: δ:2.35 (s, 3H, 4-CH_3_C_6_H_4_), 2.50 (s, 3H, 4-CH_3_-thiazole), 3.40 (dd, 1H, *J *= 13.6 Hz, 16.2 Hz, pyrazoline-H), 3.83 (dd, 1H, *J *= 13.6 Hz, 16.2 Hz, pyrazoline-H), 5.60 (dd, 1H, *J *= 13.6 Hz, 16.2 Hz, pyrazoline-H), 6.36–8.60 (m, 7H, ArH’s and furyl protons); ^13^C-NMR (DMSO-*d*6) δ:17.0 (CH_3_), 21.4 (CH_3_), 35.7 (CH_2_), 61.8 (CH), 94.5, 107.8, 110.6, 112.9, 125.7, 129.3, 130.0, 140.7, 142.4, 146.2, 148.9, 151.1, 154.0, 166.4. MS (*m/z*): 393 (M+ 1, 4), 392 (M+, 14), 206 (19), 205 (100), 190 (13), 161 (17), 127 (9), 103 (11), 86 (11); *Anal.* Calcd. for C_19_H_16_N_6_O_2_S (392.43): C, 58.15; H, 4.11; N, 21.42; S, 8.17; found: C, 58.17; H, 4.10; N, 21.42; S, 8.16.

##### Compound (**7**). Additional file 7: Figure S7

Amixture of 2-(5-(furan-2-yl)-3-(*p*-tolyl)-4,5-dihydro-1*H*-pyrazol-1-yl)-4-methylthiazole-5-carbohydrazide (5) (3.81 g, 10 mmol), ammonium thiocyanate (5 g, 6.5 mmol) and hydrochloric acid (50 mL, 37% 150 mL of H_2_O) was heated under reflux for 1 h.The resulting oily residue was solidified, collected, and recrystallized from glacial acetic acid to obtain a white solid, yield (1.49 g, 34%), mp: 230–232 °C; IR (KBr, cm^−1^): 3268 (N–H), 3150 (=C–H aromatic), 3037 (=C–H), 2966 (–C–H), 1666 (–C=O); ^1^H NMR: δ:2.36 (s, 3H, 4-CH_3_C_6_H_4_), 2.42 (s, 3H, 4-CH_3_-thiazole), 3.48 (dd, 1H, *J *= 13.6 Hz, 16.2 Hz, pyrazoline-H), 3.88 (dd, 1H, *J *= 13.6 Hz, 16.2 Hz, pyrazoline-H), 5.76 (dd, 1H, *J *= 13.6 Hz, 16.2 Hz, pyrazoline-H), 6.39-7.69 (m, 9H, ArH’s, furyl-H’s and 2 N–H), 9.23 (s, 1H, N–H), 9.58 (s, 1H, N-H); ^13^C-NMR (DMSO-*d*6) δ:17.0 (CH_3_), 21.4 (CH_3_), 35.7 (CH_2_), 61.8 (CH), 94.5, 108.8, 110.6, 125.7, 129.2, 130.0, 140.7, 142.2, 145.2, 149.8, 151.0, 154.1, 157.8, 181.2. MS (*m/z*): 440 (M+, 2), 438 (9), 425 (14), 382 (18), 319 (22), 318 (100), 290 (33), 205 (11), 169 (10), 151 (19), 128 (14); *Anal.* Calcd. for C_20_H_20_N_6_O_2_S_2_ (440.54): C, 54.53; H, 4.58; N, 19.08; S, 14.56; found: C, 54.55; H, 4.57; N, 19.08; S, 14.55.

### Compounds (**10a**, **b**) and (**11a**, **b**), General procedure

A mixture of 2-(5-(furan-2-yl)-3-(*p*-tolyl)-4,5-dihydro-1*H*-pyrazol-1-yl)-4-methylthiazole-5-carbohydrazide (**5**) (3.81 g, 10 mmol), and the appropriate amount of 3-(2-arylhydrazono)pentane-2,4-dione or ethyl 3-oxo-2-(2-arylhydrazono)butanoate (10 mmol) in acetic acid (20 mL) was heated under reflux for 2 h. The reaction mixture was left to cool to room temperature. The formed solid was filtered off, dried, and recrystallized from an appropriate solvent to obtain the corresponding compounds (**10a**, **10b**, **11a**, and **11d**), respectively.

#### Compound (**10a**). Additional file 8: Figure S8

Yellow solid from glacial acetic acid, yield (3.90 g, 71%), mp: 234–235 °C; IR (KBr, cm^−1^): 3432 (N-H), 3112 (=C–H aromatic), 2965 (–C–H), 1699 (C=O); ^1^H NMR: δ:2.42 (s, 3H, 4-CH_3_C_6_H_4_), 2.62 (s, 3H, pyrazole–CH_3_), 2.74 (s, 3H, pyrazole–CH_3_), 3.02 (s, 3H, 4-CH_3_-thiazole), 3.62 (dd, 1H, *J *= 13.6 Hz, 16.2 Hz, pyrazoline-H), 3.69 (dd, 1H, *J *= 13.6 Hz, 16.2 Hz, pyrazoline-H), 5.85 (dd, 1H, *J *= 13.6 Hz, 16.2 Hz, pyrazoline-H), 6.32 (q, 1H, Furyl-H), 6.45 (d, 1H, Furyl-H), 7.25–7.86 (m, 10H, ArH’s, 1Furyl-H); ^13^C-NMR (DMSO-*d*6) δ:11.4 (CH_3_), 12.28 (CH_3_), 17.0 (CH_3_), 21.4 (CH_3_), 35.7 (CH_2_), 61.8 (CH), 94.6, 109.6, 110.6, 121.7, 125.7, 129.2, 130.0, 130.2, 136.0, 138.7, 148.6, 150.9, 151.4, 152.4, 153.9, 160.2. MS (*m/z*): 549 (M + , 4), 515 (19), 431 (10), 430 (57), 304 (14), 132 (16), 128 (59), 127 (45), 89 (10), 88 (15), 62 (20), 61 (22), 43 (100); *Anal.* Calcd. for C_30_H_27_N_7_O_2_S (549.65): C, 65.56; H, 4.95; N, 17.84; S, 5.83; found: C, 65.59; H, 4.94; N, 17.85; S, 5.80.

#### Compound (**10b**). Additional file 9: Figure S9

Yellow solid from glacial acetic acid, yield (4.17 g, 74%), mp: 225–226 °C; IR (KBr, cm^−1^): 3107 (=C–H aromatic), 3025 (=C–H), 2972 (–C–H), 1670 (C=O); ^1^H NMR: δ: 2.42 (s, 3H, 4-CH_3_C_6_H_4_), 2.43 (s, 3H, 4-CH_3_C_6_H_4_), 2.6 (s, 3H, pyrazole–CH_3_), 2.74 (s, 3H, pyrazole–CH_3_), 3.01 (s, 3H, 4-CH_3_-thiazole), 3.63 (dd, 1H, *J *= 13.6 Hz, 16.2 Hz, pyrazoline-H), 3.70 (dd, 1H, *J *= 13.6 Hz, 16.2 Hz, pyrazoline-H), 5.80 (dd, 1H, *J *= 13.6 Hz, 16.2 Hz, pyrazoline-H), 6.32–7.77 (m, 11H, ArH’s, furyl-H’s); ^13^C-NMR (DMSO-*d*6) δ:11.4 (CH_3_), 12.28 (CH_3_), 17.0 (CH_3_), 21.4 (CH_3_), 35.7 (CH_2_), 61.8 (CH), 94.6, 109.6, 110.6, 119.2, 125.8, 129.3, 129.7, 130.0, 130.1, 138.7, 139.1. 140.8, 141.7, 142.5, 149.3, 149.9, 150.9, 151.3, 153.9, 160.2. MS (*m/z*): 565 (M+ 2, 2), 564 (M+ 1, 15), 563 (M+, 59), 522 (24), 450 (16), 432 (34), 431 (100), 327 (23), 326 (88), 296 (12), 91 (12); *Anal.* Calcd. for C_31_H_29_N_7_O_2_S (563.67): C, 66.05; H, 5.19; N, 17.39; S, 5.69; found: C, 66.07; H, 5.18; N, 17.39; S, 5.68.

#### Compound (11a). Additional file 10: Figure S10

Orange solid from dioxane, yield (3.69 g, 67%), mp: 279–280 °C; IR (KBr, cm^−1^): 3431 (O–H), 3141 (=C–H aromatic), 3067 (=C–H aromatic), 2918 (–C–H), 1701 (C=O); ^1^H NMR: δ: 2.40 (s, 3H, 4-CH_3_C_6_H_4_), 2.41 (s, 3H, 4-CH_3_-thiazole), 2.70 (s, 3H, pyrazole–CH_3_), 3.62 (dd, 1H, *J *= 13.6 Hz, 16.2 Hz, pyrazoline-H), 3.71 (dd, 1H, *J *= 13.6 Hz, 16.2 Hz, pyrazoline-H), 5.90 (dd, 1H, *J *= 13.6 Hz, 16.2 Hz, pyrazoline-H), 6.31–7.73 (m, 12H, ArH’s, furyl-H’s), 13.58 (s, 1H, O–H); ^13^C-NMR (DMSO-*d*6) δ:11.4 (CH_3_), 12.28 (CH_3_), 17.0 (CH_3_), 21.4 (CH_3_), 35.7 (CH_2_), 61.8 (CH), 94.6, 109.6, 110.6, 119.2, 125.7. 127.3, 129.7, 130.1, 130.2. 138.7, 139.2, 140.7141.7, 142.3, 149.2, 149.9, 151.0, 151.4, 154.1, 160.3. MS (*m/z*): 551 (M+, 1), 501 (10), 398 (11), 236 (25), 235 (100), 155 (10), 91 (11), 18 (22); *Anal.* Calcd. for C_29_H_25_N_7_O_3_S (551.62): C, 63.14; H, 4.57; N, 17.77; S, 5.81; found: C, 63.17; H, 4.56; N, 17.76; S, 5.80.

#### Compound (**11b**). Additional file 11: Figure S11

Orange solid from dioxane, yield (4.52 g, 80%), mp. 289–290 °C; IR (KBr, cm^−1^): 3437 (OH), 3143(=C–H aromatic), 3064 (=C–H aromatic), 2918 (–C–H), 1699 (C=O); ^1^H NMR: δ: 2.87 (s, 6H, 4-CH_3_C_6_H_4_), 2.94 (s, 3H, pyrazole–CH_3_), 2.96 (s, 3H, 4-CH_3_-thiazole), 3.57 (dd, 1H, *J *= 13.6 Hz, 16.2 Hz, pyrazoline-H), 3.62 (dd, 1H, *J *= 13.6 Hz, 16.2 Hz, pyrazoline), 5.96 (dd, 1H, *J *= 13.6 Hz, 16.2 Hz, pyrazoline-H), 6.31–8.02 (m, 11H, ArH’s, furyl-H’s), 13.62 (s, 1H, N–H); ^13^C-NMR (DMSO-*d*6) δ: 11.0 (CH_3_), 17.0 (CH_3_), 20.8 (CH_3_), 21.49 (CH_3_), 35.7 (CH_2_), 61.8 (CH), 94.6, 109.6, 110.6, 119.2, 125.7, 127.3, 129.7, 130.1, 130.2, 138.7, 139.2, 140.7, 141.7, 142.3, 149.2, 149.9, 151.0, 151.4, 154.1, 160.3. MS (*m/z*): 567 (M+ 2, 11), 566 (M+ 1, 46), 565 (M+ , 100), 425 (12), 385 (15), 215 (5), 179 (5), 105 (6), 95 (6), 91 (6), 55 (10), 43 (16); *Anal.* Calcd. for C_30_H_27_N_7_O_3_S (565.65): C, 63.70; H, 4.81; N, 17.33; S, 5.67; found: C, 63.73; H, 4.80; N, 17.30; S, 5.67.

### Compounds (**12a** and **12b**), and (**13**), general procedure

A mixture of 2-(5-(furan-2-yl)-3-(*p*-tolyl)-4,5-dihydro-1*H*-pyrazol-1-yl)-4-methylthiazole-5-carbonyl azide (**6**) (2.2 g, 5 mmol) and the appropriate amount of aromatic amines (aniline, 4-methylaniline), anthranilic acid or methyl anthranilate (5 mmol) in dioxane (20 mL), was heated under reflux for 3 h. The reaction mixture was left to cool to room temperature. The formed solid formed was filtered off, dried, and recrystallized from an appropriate solvent to obtain the corresponding compounds (**12a**), and (**12b**), and (**13**), respectively.

#### Compound (**12a**). Additional file 12: Figure S12

White solid from dioxane, yield (1.83 g, 80%), mp: 226–229 °C; IR (KBr, cm^−1^): 3308 (N–H), 3104 (=C–H aromatic), 3031 (=C–H), 2918 (–C–H), 1637 (CON–H); ^1^H NMR: δ: 2.06 (s, 3H, 4-CH_3_C_6_H_4_), 2.35 (s, 3H, 4-CH_3_-thiazole), 3.45 (dd, 1H, *J *= 13.6 Hz, 16.2 Hz, pyrazoline-H), 3.77 (dd, 1H, *J *= 13.6 Hz, 16.2 Hz, pyrazoline-H), 5.58 (dd, 1H, *J *= 13.6 Hz, 16.2 Hz, pyrazoline-H), 6.39 (s, 2H, N–H), 6.94–8.72 (m, 12H, ArH’s + furyl-H’s); ^13^C-NMR (DMSO-*d*6) δ:13.6 (CH_3_), 21.4 (CH_3_), 35.7 (CH_2_), 61.8 (CH), 94.6, 109.6, 110.6, 116.2, 125.7, 129.2, 129.7, 130.1, 134.2, 134.7, 140.7, 142.3, 148.9, 150.3, 153.9, 157.6, 160.3. MS (*m/z*): 459 (M+ 2, 1), 458 (M+ 1, 9), 257 (M+, 70), 443 (80), 278 (85), 261 (23), 260 (12), 247 (10), 181 (25), 78 (17), 79 (14), 77 (28), 75 (19), 51 (15), 43 (38), 42 (21), 41 (20), 30 (61), 28 (100); *Anal.* Calcd. for C_25_H_23_N_5_O_2_S (457.55): C, 65.63; H, 5.07; N, 15.31; S, 7.01; found: C, 65.61; H, 5.08; N, 15.32; S, 7.02.

#### Compound (**12b**). Additional file 13: Figure S13

Pale yellow solid from dioxane, yield (1.62 g, 75%), mp: 191–192 °C; IR (KBr, cm^−1^): 3308 (N–H), 3104 (=C–H aromatic), 3031 (=C–H), 2918 (–C–H), 1637 (CONH); ^1^H NMR: δ:2.06 (s, 6H, 2CH_3_C_6_H_4_), 2.35 (s, 3H, 4–CH_3_-thiazole), 3.45 (dd, 1H, *J *= 13.6 Hz, 16.2 Hz, pyrazoline-H), 3.77 (dd, 1H, *J *= 13.6 Hz, 16.2 Hz, pyrazoline-H), 5.58 (dd, 1H, *J *= 13.6 Hz, 16.2 Hz, pyrazoline-H), 6.39 (s, 2H, N–H), 6.94–8.72 (m, 11H, ArH’s + furyl-H’s); ^13^C-NMR (DMSO-*d*6) δ:13.6 (CH_3_), 20.8 (CH_3_), 21.4 (CH_3_), 35.7 (CH_2_), 61.8 (CH), 94.6, 109.6, 110.6, 112.1, 125.7, 129.1, 129.2, 130.0, 134.1, 140.7, 142.4, 148.5. 150.9, 154.0, 157.6, 160.4; *Anal.* Calcd. for C_26_H_25_N_5_O_2_S (471.58): C, 66.22; H, 5.34; N, 14.85; S, 6.80; found: C, 66.11; H, 545; N, 14.98; S, 6.69.

#### Compound (**13**). Additional file 14: Figure S14

White solid from glacial acetic acid, yield (1.71 g, 71%), mp: 260–263 °C; IR (KBr, cm^−1^): 3286 (N–H), 3157 (=C–H aromatic), 2955 (–C–H), 1735 (–C=O), 1657 (CON–H); ^1^H NMR: δ: 2.34 (s, 3H, 4-CH_3_C_6_H_4_), 2.35 (s, 3H, 4-CH_3_-thiazole), 3.44 (dd, 1H, *J *= 13.6 Hz, 16.2 Hz, pyrazoline-H), 3.84 (dd 1H, *J *= 13.6 Hz, 16.2 Hz, pyrazoline-H), 5.76 (dd, 1H, *J *= 13.6 Hz, 16.2 Hz, pyrazoline-H), 6.44 (m, 2H, furyl-H’s), 7.20–7.95 (m, 9H, ArH’s + 1Furyl-H), 11.58 (s, 1H, N–H); ^13^C-NMR (DMSO-*d*6) δ:13.7 (CH_3_), 21.4 (CH_3_), 35.7 (CH_2_), 61.8 (CH), 94.6, 110.6, 114.6, 115.2, 117.3, 123.0, 125.7, 127.9, 129.3, 130.0, 139.8, 140.7, 142.4, 150.9, 152.1, 153.9, 156.7, 159.4, 160.8. MS (*m/z*): 483 (M+, 2%), 470 (22), 469 (85), 426 (30), 396 (23), 426 (27), 364 (18), 363 (88), 341 (27), 337 (28), 309 (40), 299 (19), 283 (34), 280 (16), 267 (65), 219 (14), 186 (37), 181 (17), 180 (34), 173 (15), 171 (93), 151 (28), 129 (24), 126 (33), 115 (32), 113 (45), 111 (30), 97 (34), 87 (24), 85 (59), 82 (25), 81 (18), 69 (35), 68 (46), 59 (3), 57 (17), 55 (24), 45 (37), 44 (32), 43 (92), 41 (38); *Anal.* Calcd. for C_26_H_21_N_5_O_3_S (483.54): C, 64.58; H, 4.38; N, 14.48; S, 6.63; found: C, 64.54; H, 4.39; N, 14.49; S, 6.65.

#### Compound (**14**). Additional file 15: Figure S15

Amixture of 2-(5-(furan-2-yl)-3-(*p*-tolyl)-4,5-dihydro-1*H*-pyrazol-1-yl)-4-methylthiazole-5-carbonyl azide (**6**) (2.2 g, 5 mmol), and 2-naphthol (0.72 g, 5 mmol), in dry benzene (20 mL) was refluxed for 3 h. The reaction mixture was left to cool at room temperature. The formed solid was filtered off, dried, and recrystallized from glacial acetic acid to obtain compound (**14**) as a brown solid, yield (2.11 g, 83%), mp: 219–222 °C; IR (KBr, cm^−1^): 3286 (N–H), 3157 (=C–H aromatic), 2955 (–C–H), 1735 (–C=O), ^1^H NMR: δ: 2.14 (s, 3H, 4-CH_3_C_6_H_4_), 2.35 (s, 3H, 4–CH_3_-thiazole), 3.37 (dd, 1H, *J *= 13.6 Hz, 16.2 Hz, pyrazoline-H), 3.81 (dd, 1H, *J *= 13.6 Hz, 16.2 Hz, pyrazoline-H), 5.61 (dd, 1H, *J *= 13.6 Hz, 16.2 Hz, pyrazoline-H), 6.40–8.13 (m, 14H, ArH’s, furyl-H’s), 8.95 (s, 1H, N-H); ^13^C-NMR (DMSO-*d*6) δ:17.7 (CH_3_), 21.4 (CH_3_), 35.7 (CH_2_), 61.8 (CH), 94.6, 110.6, 116.9, 117.3, 118.9, 125.7, 126.4, 127.6, 127.8, 129.2, 129.3, 134.1, 14.7, 172.4, 149.8, 151.0, 151.6, 152.9, 155.0, 160.1. MS (*m/z*): 508 (M+, 2), 307 (100), 201 (14), 172 (13), 171 (26), 156 (26), 132 (32), 128 (19), 106 (21), 105 (29), 104 (27); *Anal.* Calcd. for C_29_H_24_N_4_O_3_S (508.59): C, 68.49; H, 4.76; N, 11.02; S, 6.30; found: C, 68.48; H, 4.75; N, 11.00; S, 6.32.

#### Compound (**15**). Additional file 16: Figure S16

2-(5-(Furan-2-yl)-3-(*p*-tolyl)-4,5-dihydro-1*H*-pyrazol-1-yl)-4-methylthiazole-5-carbohydrazide (3.81 g, 10 mmol) was suspended in ethanol, and then carbon disulfide (10 mL) was added, dropwise, to the suspension at 5–10 °C. The mixture was heated for 10 h under reflux in the presence of potassium hydroxide (0.56 g, 10 mmol). The solution was cooled and acidified to pH 5–6 using HCl solution, and the formed solid was collected and recrystallized to obtain a yellow solid from dioxane, yield (3.05 g, 72%), mp: 267–270 °C; IR (KBr, cm^−1^): 3110 (S–H), 2920 (–C–H); ^1^H NMR: δ: 2.36 (s, 3H, 4-CH_3_C_6_H_4_), 2.41 (s, 3H, 4-CH_3_-thiazole), 3.50 (dd, 1H, *J *= 13.6 Hz, 16.2 Hz, pyrazoline-H), 3.92 (dd, 1H, *J *= 13.6 Hz, 16.2 Hz, pyrazoline-H), 5.80 (dd, 1H, *J *= 13.6 Hz, 16.2 Hz, pyrazoline-H), 6.41–7.72 (m, 7H, ArH’s + furyl-H’s), 14.55 (s, 1H, S–H); ^13^C-NMR (DMSO-*d*6) δ:17.5 (CH_3_), 21.5 (CH_3_), 35.7 (CH_2_), 61.8 (CH), 94.6, 110.6, 125.7, 129.3, 130.1, 140.7, 140.9, 142.4, 149.8, 150.9, 152.7, 152.9, 169.2. MS (*m/z*): 424 (M+ 1, 4), 423 (M+, 5), 392 (20), 230 (8), 216 (12), 192 (13), 190 (15), 189 (100); *Anal.* Calcd. for C_20_H_17_N_5_O_2_S_2_ (423.51): C, 56.72; H, 4.05; N, 16.54; S, 15.14; found: C, 56.74; H, 4.04; N, 16.55; S, 15.12.

### Compounds (**20a**–**d**), General procedure

Equal molar quantities of 5-(2-(5-(furan-2-yl)-3-(*p*-tolyl)-4,5-dihydro-1*H*-pyrazol-1-yl)-4-methylthiazol-5-yl)-1,3,4-oxadiazole-2-thiol (**15**) (2.11 g, 5 mmol), and the appropriate hydrazonoyl halides (**16a**–**d**) (5 mmol) in ethanol (20 mL) containing a catalytic amount of triethylamine were heated under reflux for 2 h. The reaction mixture was left to cool to room temperature. The formed solid was filtered off, dried, and recrystallized from an appropriate solvent to obtain the corresponding compounds (**20a**–**d**), respectively.

#### Compound (**20a**). Additional file 17: Figure S17

Yellow solid from glacial acetic acid, yield (2.36 g, 77%), mp: 206–209 °C; IR (KBr, cm^−1^): 3438 (N–H), 3153; 3037 (=C–H), 2973; 2925 (–C–H), 1703 (–C=O); ^1^H NMR: δ: 1.31 (t, 3H, –OCH_2_CH_3_), 2.37 (s, 3H, 4-CH_3_C_6_H_4_), 2.42 (s, 3H, 4-CH_3_-thiazole), 3.47(dd, 1H, *J *= 13.6 Hz, 16.2 Hz, pyrazoline-H), 3.83 (dd 1H, *J *= 13.6 Hz, 16.2 Hz, pyrazoline-H), 3.83 (dd, 1H, *J *= 13.6 Hz, 16.2 Hz, pyrazoline-H), 4.33 (q, 2H, –OCH_2_CH_3_), 6.41 (m, 2H, furyl-H’s), 7.30–7.90 (m, 10H, ArH’s, 1Furyl-H), 10.54 (s, 1H, N–H); ^13^C-NMR (DMSO-*d*6) δ:13.9 (CH_3_), 17.0 (CH_3_), 21.4 (CH_3_), 35.7 (CH_2_), 61.8 (CH), 62.6, 94.6, 107.9, 110.6, 123.1, 125.7, 130.1, 129.2, 129.3, 138.8, 140.7, 142.4, 146.6, 147.9, 149.9, 151.1, 154.0, 154.2, 131.3, 161.4. MS (*m/z*): 613 (M+, 9), 609 (11), 409 (10), 406 (13), 390 (22), 360 (12), 239 (14), 168 (13), 152 (59), 151 (100), 135 (29), 129 (11), 106 (17), 85 (30), 73 (30), 71 (50), 69 (25), 55 (38), 43 (82), 29 (17); *Anal.* Calcd. for C_30_H_27_N_7_O_4_S_2_ (613.71): C, 58.71; H, 4.43; N, 15.98; S, 10.45; found: C, 58.73; H, 4.41; N, 15.99; S, 10.44.

#### Compound (**20b**). Additional file 18: Figure S18

Yellow solid from glacial acetic acid, yield (1.89 g, 65%), mp: 258–261 °C; IR (KBr, cm^−1^): 3430 (N–H), 3160; 3109 (=C–H), 2925 (–C–H), 1679 (–C=O); ^1^H NMR: δ: 2.37 (s, 3H, CO–CH_3_), 2.43 (s, 3H, 4-CH_3_C_6_H_4_), 2.50 (s, 3H, 4-CH_3_-thiazole), 3.40 (dd, 1H, *J *= 13.6 Hz, 16.2 Hz, pyrazoline-H), 3.83 (dd, 1H, *J *= 13.6 Hz, 16.2 Hz, pyrazoline-H), 5.74 (dd, 1H, *J *= 13.6 Hz, 16.2 Hz, pyrazoline-H), 6.41 (m, 2H, furyl-H’s), 7.30–7.97 (m, 10H, ArH’s, 1Furyl-H), 10.52 (s, 1H, N–H); ^13^C-NMR (DMSO-*d*6) δ:17.7 (CH_3_), 21.4 (CH_3_), 24.8 (CH_3_), 35.7 (CH_2_), 61.8 (CH), 94.6, 107.9, 110.6, 116.9, 123.1, 125.7, 129.1, 129.2, 130.0, 140.7, 142.4, 146.6, 149.8, 150.2. 150.9, 152.9, 154.4, 161.3, 191. MS (*m/z*): 583 (M + , 9), 515 (19), 430 (56), 304 (13), 132 (15), 128 (59), 127 (45), 61 (22), 43 (100); *Anal.* Calcd. for C_29_H_25_N_7_O_3_S_2_ (583.68): C, 59.67; H, 4.32; N, 16.80; S, 10.99; found: C, 59.66; H, 4.33; N, 16.81; S, 10.98.

#### Compound (**20c**). Additional file 19: Figure S19

Red solid from dioxane, yield (2.00 g, 62%) mp: 255–256 °C; IR (KBr, cm^−1^): 3245 (N–H), 3130 (=C–H), 2963 (–C–H), 1617 (–C=O); ^1^H NMR: δ: 2.37 (s, 3H, 4-CH_3_C_6_H_4_), 2.45 (s, 3H, 4-CH_3_-thiazole), 3.47 (dd, 1H, *J *= 13.6 Hz, 16.2 Hz, pyrazoline-H), 3.84 (dd, 1H, *J *= 13.6 Hz, 16.2 Hz, pyrazoline-H), 5.75 (dd, 1H, *J *= 13.6 Hz, 16.2 Hz, pyrazoline-H), 6.41 (m, 2H, furyl-H’s), 7.31–8.23 (m, 15H, ArH’s, 1Furyl-H), 10.57 (s, 1H, N–H); ^13^C-NMR (DMSO-*d*6) δ:17.0 (CH_3_), 21.4 (CH_3_), 35.7 (CH_2_), 61.8 (CH), 94.6, 107.9, 110.6, 123.1, 125.7, 128.3, 140.7, 142.4, 146.4, 149.9, 150.4, 153.9, 154.2, 155.9, 161.3. MS (*m/z*): 645 (M + , 1), 498 (11), 339 (11), 281 (17), 243 (51), 242 (11), 256 (12), 239 (15), 153 (15), 152 (60), 151 (100), 135 (29), 106 (17), 85 (31), 83 (62), 171 (32), 73 (35), 71 (76), 60 (100), 43 (51); *Anal.* Calcd. for C_34_H_27_N_7_O_3_S_2_ (645.75): C, 63.24; H, 4.21; N, 15.18; S, 9.93; found: C, 63.27; H, 4.20; N, 15.16; S, 9.93.

#### Compound (**20d**). Additional file 20: Figure S20

Yellow solid from dioxane, yield (2.34 g, 71%), mp: 244-247 °C; IR (KBr, cm^−1^): 3245 (N–H), 3130 (=C–H), 2963 (–C–H), 1667 (–C=O); ^1^H NMR δ: 2.37 (s, 3H, 4-CH_3_C_6_H_4_), 2.45 (s, 3H, 4-CH_3_-thiazole), 3.47 (dd, 1H, *J *= 13.6 Hz, 16.2 Hz, pyrazoline-H), 3.83 (dd, 1H, *J *= 13.6 Hz, 16.2 Hz, pyrazoline-H), 5.75 (dd, 1H, *J *= 13.6 Hz, 16.2 Hz, pyrazoline-H), 6.40 (m, 2H, furyl-H’s), 7.14–8.13 (m, 15H, ArH’s + 1Furyl-H), 10.55 (s, 1H, N–H), 107.9 (s, 1H, N–H); ^13^C-NMR (DMSO-*d*6) δ:17.1 (CH_3_), 21.4 (CH_3_), 35.7 (CH_2_), 61.8 (CH), 94.6, 110.6, 121.1, 125.7, 128.5, 129.1, 130.7, 137.1, 138.8, 140.7, 142.4, 146.4, 147.9, 149.8, 151., 153.9, 161.3. MS (*m/z*): 660 (M + , 10), 382 (13), 359 (10), 341 (66), 340 (18), 284 (23), 268 (19), 267 (100), 185 (20), 129 (35), 116 (25), 112 (25), 109 (15), 98 (80), 84 (37), 83 (41), 55 (50), 43 (63); *Anal.* Calcd. for C_34_H_28_N_8_O_3_S_2_ (660.77): C, 61.80; H, 4.27; N, 16.96; S, 9.71; found: C, 61.84; H, 4.25; N, 16.95; S, 9.70.

### Compounds (**21a**) and (**21b**), general procedure

A mixture of 2-(2-(5-(furan-2-yl)-3-(*p*-tolyl)-4,5-dihydro-1*H*-pyrazol-1-yl)-4-methylthiazole-5-carbonyl)hydrazinecarbothioamide (**7**) (2.20 g, 5 mmol), and the appropriate hydrazonoyl halides (**16b** and **16c**) (5 mmol), in ethanol (20 mL) containing a catalytic amount of triethylamine was heated under reflux for 2 h. The reaction mixture was left to cool to room temperature. The formed solid was filtered off, dried, and recrystallized from glacial acetic acid to obtain compounds (**21a**), and (**21b**), respectively.

#### Compound (**21a**). Additional file 21: Figure S21

Red solid from glacial acetic acid, yield (1.51 g, 52%), mp: 239–240 °C; IR (KBr, cm^−1^): 3432 (N–H), 3034 (=C–H), 2922 (–C–H), 1625 (C=O); ^1^H NMR: δ: 2.37 (s, 3H, 4-CH_3_C_6_H_4_), 2.49 (s, 3H, 4-CH_3_-thiazole), 2.49 (s, 3H, 4-CH_3_-thiazole), 3.43 (dd, 1H, *J *= 13.6 Hz, 16.2 Hz, pyrazoline-H), 3.85 (dd, 1H, *J *= 13.6 Hz, 16.2 Hz, pyrazoline-H), 5.75 (dd, 1H, *J *= 13.6 Hz, 16.2 Hz, pyrazoline-H), 6.41–7.72 (m, 13H, ArH’s + 1 N–H, furyl-H’s), 10.51 (s, 1H, N–H); ^13^C-NMR (DMSO-*d*6) δ:13.4 (CH_3_),17.1 (CH_3_), 21.4 (CH_3_), 35.7 (CH_2_), 61.8 (CH), 49.5, 108.8, 110.6, 122.3, 129.3, 125.6, 140.4, 142.4, 146.4, 145.2, 149.8, 150.0., 152.4, 153.9, 157.8, 171.6. MS (*m/z*): 582 (M + 1, 3), 581 (M + , 65), 301 (13), 300 (33), 299 (100), 298 (12), 288 (12), 287 (16), 28 6(78), 285 (11), 239 (19), 227 (25), 225 (15), 211 (18), 44 (31), 18 (17); *Anal.* Calcd. for C_29_H_26_N_8_O_2_S_2_ (582.70): C, 59.78; H, 4.50; N, 19.23; S, 11.01; found: C, 59.80; H, 4.49; N, 19.21; S, 11.00.

#### Compound (**21b**). Additional file 22: Figure S22

Red solid from glacial acetic acid, yield (1.45 g, 45%), mp: 227–230 °C; IR (KBr, cm^−1^): 3434 (N–H), 3022 (=C–H), 2918 (–C–H), 1631 (CON–H); ^1^H NMR: δ: 2.37 (s, 3H, 4-CH_3_C_6_H_4_), 2.49 (s, 3H, 4-CH_3_-thiazole), 3.50 (dd, 1H, *J *= 13.6 Hz, 16.2 Hz, pyrazoline-H), 3.87 (dd, 1H, *J *= 13.6 Hz, 16.2 Hz, pyrazoline-H), 5.79 (dd, 1H, *J *= 13.6 Hz, 16.2 Hz, pyrazoline-H), 6.41–8.26 (m, 18H, ArH’s, 1 N-H, furyl-H’s), 10.73 (s, 1H, N-H); ^13^C-NMR (DMSO-*d*6) δ:17.0 (CH_3_), 21.4 (CH_3_), 35.7 (CH_2_), 61.8 (CH), 94.6, 108.6, 110.6, 122.3, 125.7, 129.0, 129.1, 129.9, 134.5, 138.9, 140.7, 142.4, 145.3, 147.4, 149.8, 151. 0, 152.4, 157.7, 170.3. MS (*m/z*): 644 (M + , 8), 614 (11), 607 (9), 308 (10), 281 (17), 243 (51), 242 (63), 210 (13), 170 (13), 156 (25), 73 (35), 71 (76), 60 (100), 55 (18), 43 (51), 41 (26); *Anal.* Calcd. for C_34_H_28_N_8_O_2_S_2_ (644.77): C, 63.33; H, 4.38; N, 17.38; S, 9.95; found: C, 63.36; H, 4.37; N, 17.37; S, 9.94.

### Compounds (**22**) and (**23**), general procedure

A mixture of 2-(5-(furan-2-yl)-3-(*p*-tolyl)-4,5-dihydro-1*H*-pyrazol-1-yl)-4-methylthiazole-5-carbohydrazide (**5**) (1.95 g, 5 mmol), and the appropriate maleic anhydride or phthalic anhydride (5 mmol) was heated under reflux in glacial acetic acid for 2 h. The reaction mixture was left to cool to room temperature. The formed solid was filtered off, dried, and recrystallized from acetic acid to obtain compounds (**22**) and (**23**), respectively.

#### Compound (**22**). Additional file 23: Figure S23

Yellow solid, yield (1.93 g, 84%), mp: 230–233 °C; IR (KBr, cm^−1^): 3398; 3229 (N-H), 2951 (–C–H), 1715 (–C=O); ^1^H NMR: δ: 2.36 (s, 3H, 4-CH_3_C_6_H_4_), 2.41 (s, 3H, 4-CH_3_-thiazole), 3.50 (dd, 1H, *J *= 13.6 Hz, 16.2 Hz, pyrazoline-H), 3.91 (dd, 1H, *J *= 13.6 Hz, 16.2 Hz, pyrazoline-H), 5.77 (dd, 1H, *J *= 13.6 Hz, 16.2 Hz, pyrazoline-H), 6.26–7.70 (m, 10H, Ar–H, furyl-H’s, pyridazine-H); ^13^C-NMR (DMSO-*d*6) δ:17.0 (CH_3_), 21.4 (CH_3_), 35.7 (CH_2_), 61.6 (CH), 94.6, 110.2, 110.6, 125.2, 125.7, 129.5, 140.7, 142.4, 149.4, 149.8, 151., 157.1, 158.8, 167.1. MS (*m/z*): 461 (M + , 9), 402 (20), 384 (100), 369 (41), 351 (29), 247 (20), 144 (14), 230 (11), 159 (18), 149 (16), 145 (18), 135 (25), 133 (17) 122 (17), 121 (22), 105 (21), 95 (38), 91 (18), 67 (18), 57 (22), 55 (31), 43 (40); *Anal.* Calcd. for C_23_H_19_N_5_O_4_S (461.49): C, 59.86; H, 4.15; N, 15.18; S, 6.95; found: C, 59.89; H, 4.14; N, 15.17; S, 6.94.

#### Compound (**23**). Additional file 24: Figure S24

White solid, yield (1.63 g, 64%), mp: 152–154 °C; IR (KBr, cm^−1^): 3436 (N–H), 2923 (–C–H), 1735 (–C=O); ^1^H NMR: δ: 2.41 (s, 3H, 4-CH_3_C_6_H_4_), 2.58 (s, 3H, 4-CH_3_-thiazole), 3.65 (dd, 1H, *J *= 13.6 Hz, 16.2 Hz, pyrazoline-H), 3.71 (dd, 1H, *J *= 13.6 Hz, 16.2 Hz, pyrazoline-H), 5.80 (dd, 1H, *J *= 13.6 Hz, 16.2 Hz, pyrazoline-H), 6.32 (q, 1H, Furyl-H), 6.40 (d, 1H, furyl-H), 7.24–7.94 (m, 10H, ArH’s, 1Furyl-H, N–H); ^13^C-NMR (DMSO-*d*6) δ:17.1 (CH_3_), 21.4 (CH_3_), 35.7 (CH_2_), 61.8 (CH), 94.6, 106, 110.6, 125.7, 129.2, 132.7, 140.7, 142.4, 149.5, 149.8, 150.9., 153.8, 155.8, 163.8. MS (*m/z*): 511 (M + , 31), 453 (26), 452 (53), 437 (13), 263 (17), 262 (69), 250 (20), 249 (51), 248 (22), 203 (36), 202 (21), 191 (25), 189 (100) 188 (21), 187 (28), 175 (31), 136 (25), 135(25), 119 (26), 107 (27), 105 (21), 95 (29), 93 (26), 81 (34), 69 (34); *Anal.* Calcd. for C_27_H_21_N_5_O_4_S (511.55): C, 63.39; H, 4.14; N, 13.69; S, 6.27; found: C, 63.41; H, 4.14; N, 13.68; S, 6.26.

### Compounds (**25a**–**c**), general methods

*Method A* A mixture of 2-(5-(furan-2-yl)-3-(*p*-tolyl)-4,5-dihydro-1*H*-pyrazol-1-yl)thiazol-5(4*H*)-one (**2**) (1.6 g, 5 mmol), and the appropriate arylidenemalononitrile (**24a**–**c**) in ethanol (20 mL) containing a catalytic amount of piperdine was heated under reflux for 2 h. The reaction mixture was left to cool to room temperature. The formed solid was filtered off, dried, and recrystallized from dioxane to yield compounds (**25a**–**c**), respectively.

*Method B* A mixture of compound (**2**) (1.6 g, 5 mmol) and the corresponding amount of benzaldehyde, 4-methylbenzaldehyde or 4-methoxybenzaldehyde (5 mmol), malononitrile (0.33 g, 5 mmol), and piperdine (0.42 g, 5 mmol) in ethanol (20 mL) was heated for 2 h under reflux. The formed solid was filtered off, dried, and recrystallized from dioxane to obtain products that were identical in all respects (mp, mixed mp, and IR spectra) to the product obtained using Method A.

#### Compound (**25a**). Additional file 25: Figure S25

White solid from dioxane, yield (2.03 g, 85%), mp: 250–252 °C; IR (KBr, cm^−1^): 3388; 3262 (N–H), 3158 (–C=H), 2925 (–C–H), 2100 (–CN); ^1^H NMR: δ: 2.35 (s, 3H, 4-CH_3_C_6_H_4_), 4.20 (s, 1H, pyran-H), 3.28 (dd, 1H, *J *= 13.6 Hz, 16.2 Hz, pyrazoline-H), 3.70 (dd, 1H, *J *= 13.6 Hz, 16.2 Hz, pyrazoline-H), 5.96 (dd, 1H, *J *= 13.6 Hz, 16.2 Hz, pyrazoline-H), 6.28 (d, 1H, Furyl-H), 6.37 (q, 1H, Furyl-H), 7.26–7.79 (m, 10H, ArH’s, 1furyl-H), 7.97 (s, 2H, -NH_2_); ^13^C-NMR (DMSO-*d*6) *δ*: 21.4 (CH_3_), 35.7 (CH_2_), 61.8 (CH), 66.4, 83.9, 94.6, 110.6, 118.9, 125.7, 128.3, 129.2, 130.1, 140.7, 141.4, 142.4, 150.9, 153.8., 153.9, 159.5. MS (*m/z*): 479 (M + , 9), 435 (16), 268 (21), 252 (10), 239 (16), 201 (13), 199 (11), 182 (14), 162 (23), 156 (11), 155 (12), 146 (20), 108 (23), 107 (18), 91 (100), 86 (96), 79 (23), 72 (27), 55 (12); *Anal.* Calcd. for C_27_H_21_N_5_O_2_S (479.55): C, 67.62; H, 4.41; N, 14.60; S, 6.69; found: C, 67.65; H, 4.40; N, 14.60; S, 6.67.

#### Compound (**25b**). Additional file 26: Figure S26

Yellow solid from dioxane, yield (1.90 g, 77%), mp: 196–197 °C; IR (KBr, cm^−1^): 3436 (N–H), 3035 (–C=H), 2929 (–C–H), 2150 (–CN); ^1^H NMR: δ: 2.36 (s, 3H, 4-CH_3_C_6_H_4_), 2.39 (s, 3H, 4-CH_3_C_6_H_4_), 3.30 (s, 1H, pyran-H), 3.63 (dd, 1H, *J *= 13.6 Hz, 16.2 Hz, pyrazoline-H), 3.97 (dd, 1H, *J *= 13.6 Hz, 16.2 Hz, pyrazoline-H), 5.97 (dd 1H, *J *= 13.6 Hz, 16.2 Hz, pyrazoline-H), 6.44 (q, 1H, furyl-H), 6.54 (d, 1H, furyl-H), 7.33–7.82 (m, 11H, ArH’s, 1furyl-H, -NH_2_); ^13^C-NMR (DMSO-*d*6) δ:20.9 (CH_3_), 21.1 (CH_3_), 34.1 (CH_2_), 33.7, 38.2, 61.8 (CH), 93.5, 107.9, 109.6, 128.3, 129.9, 130.2, 131.4, 125.3, 140.7, 142.8, 143.6, 148.7, 154.4, 154.8, 157.6., 159.1. MS (*m/z*): 493 (M+, 10), 492 (34), 449 (26), 377 (10), 343 (17), 333 (28), 302 (15), 297 (12), 272 (11), 270 (28), 230 (40), 229 (22), 228 (100), 200 (14), 156 (50), 104 (15), 43 (26); *Anal.* Calcd. for C_28_H_23_N_5_O_2_S (493.58): C, 68.13; H, 4.70; N, 14.19; S, 6.50; found: C, 68.16; H, 4.71; N, 14.16; S, 6.49.

#### Compound (**25c**). Additional file 27: Figure S27

Yellow solid from dioxane, yield (1.78 g, 70%), mp: 228–231 °C; IR (KBr, cm^−1^): 3436 (N–H), 3035 (–C=H), 2929 (–C–H), 2150 (–CN); ^1^H NMR: δ: 2.39 (s, 3H, 4-CH_3_C_6_H_4_), 3.62 (dd, 1H, *J *= 13.6 Hz, 16.2 Hz, pyrazoline-H), 3.83 (s, 3H, –OCH_3_), 4.01 (dd, 1H, *J *= 13.6 Hz, 16.2 Hz, pyrazoline-H), 4.70 (s, 1H, pyran-H), 5.96 (dd, 1H, *J *= 13.6 Hz, 16.2 Hz, pyrazoline-H), 6.44–7.81 (m, 13H, ArH’s, furyl-H’s, -NH_2_); ^13^C-NMR (DMSO-*d*6) δ: 21.9 (CH_3_), 33.7 (CH_2_), 34.1, 38.2, 55.2, 128.3, 128.9, 131.3. 135.2, 140.1, 142.4, 154.9, 156.1., 157.2, 159.1. MS (*m/z*): 511 (M + 2, 3), 510 (M + 2, 13), 509 (M + , 36), 407 (22), 334 (12), 256 (13), 242 (15), 233 (27), 228 (11), 156 (12), 153 (10), 105 (100), 77 (22); *Anal. Calcd*. for C_28_H_23_N_5_O_3_S (509.58): C, 66.00; H, 4.55; N, 13.74; S, 6.29; found: C, 66.02; H, 4.53; N, 13.73; S, 6.30.

## Antimicrobial activity assay

The chemical compounds being investigated were tested against a panel of Gram-positive and Gram-negative bacterial pathogens and fungi individually. Antimicrobial tests were performed using the agar well-diffusion method [50–52]. After cooling and solidifying the media, In the solidified agar, wells (6 mm in diameter) were made, the microbial inoculum was then spread evenly using a sterile cotton swab on a sterile Petri dish containing a medium of nutrient agar (NA) or Sabouraud Dextrose Agar (SDA) media for bacteria and fungi, respectively. By dissolving 1 mg of the compound in 1 mL of dimethylsulfoxide (DMSO) a 100-µL of aliquot of the tested compound solution was prepared. The inoculated plates were then incubated for bacteria and yeast for 24 h at 37 °C and fungi for 48 h at 28 °C. In order to dissolve the tested compound, the negative controls were prepared using DMSO. Amphotericin B (1 mg/mL), Ampicillin (1 mg/mL) and Gentamicin (1 mg/mL) have been used as bacterial and fungal standards, respectively. Antimicrobial activity was evaluated after incubation by measuring the inhibition zone against the microorganisms tested. Antimicrobial activity has been expressed in millimeters (mm) as inhibition diameter zones.

## Additional files

**Additional file 1: Figure S1.** ^1^H NMR, Mass and IR spectra of compound (**1**).

**Additional file 2: Figure S2.** ^1^H NMR, Mass and IR spectra of compound (**2**).

**Additional file 3: Figure S3.** ^1^H NMR, Mass and IR spectra of compound (**3**).

**Additional file 4: Figure S4.** ^1^H NMR, Mass and IR spectra of compound (**4**).

**Additional file 5: Figure S5.** ^1^H NMR, Mass and IR spectra of compound (**5**).

**Additional file 6: Figure S6.** ^1^H NMR, Mass and IR spectra of compound (**6**).

**Additional file 7: Figure S7.** ^1^H NMR, Mass and IR spectra of compound (**7**).

**Additional file 8: Figure S8.** ^1^H NMR, Mass, and IR spectra of compound (**10a**).

**Additional file 9: Figure S9.** ^1^H NMR, Mass and IR spectra of compound (**10b**).

**Additional file 10: Figure S10.** ^1^H NMR, Mass and IR spectra of compound (**11a**).

**Additional file 11: Figure S11.** ^1^H NMR, Mass, and IR spectra of compound (**11b**).

**Additional file 12: Figure S12.** ^1^H NMR, Mass and IR spectra of compound (**12a**).

**Additional file 13: Figure S13.** ^1^H NMR, Mass and IR spectra of compound (**12b**).

**Additional file 14: Figure S14.** ^1^H NMR and Mass spectra of compound (**13**).

**Additional file 15: Figure S15.** ^1^H NMR and Mass spectra of compound (**14**).

**Additional file 16: Figure S16.** ^1^H NMR, Mass and IR spectra of compound (**15**).

**Additional file 17: Figure S17.** ^1^H NMR and IR spectra of compound (**20a**).

**Additional file 18: Figure S18.** ^1^H NMR and IR spectra of compound (**20b**).

**Additional file 19: Figure S19.** ^1^H NMR and Mass spectra of compound (**20c**).

**Additional file 20: Figure S20.** ^1^H NMR spectra of compound (**20d**).

**Additional file 21: Figure S21.** ^1^H NMR and Mass spectra of compound (**21a**).

**Additional file 22: Figure S22.** ^1^H NMR, Mass and IR spectra of compound (**21b**).

**Additional file 23: Figure S23.** ^1^H NMR, Mass and IR spectra of compound (**22**).

**Additional file 24: Figure S24.** ^1^H NMR, Mass and IR spectra of compound (**23**).

**Additional file 25: Figure S25.** ^1^H NMR, Mass and IR spectra of compound (**25a**).

**Additional file 26: Figure S26.** ^1^H NMR and Mass spectra of compound (**25b**).

**Additional file 27: Figure S27.** ^1^H NMR and Mass spectra of compound (**25c**).

## Abbreviations

NA: nutrient agar
SDA: sabouraud dextrose agar
mp: melting point
Mw: molecular weight
*AF*: *Aspergillus fumigatus*
*CA*: *Candida albicans*
*SP*: *Streptococcus pneumoniae*
*BS*: *Bacillis subtillis*
*PA*: *Pseudomonas aeruginosa*
*EC*: Escherichia coli
