# Supplementary figures and images for: Utility of 5-(furan-2-yl)-3-(p-tolyl)-4,5-dihydro-1H-pyrazole-1-carbothioamide in the synthesis of heterocyclic compounds with antimicrobial activity
Source: BMC Chem. 2019 Apr 1;13(1):48. doi: 10.1186/s13065-019-0566-y (PMC6661803; doi:10.1186/s13065-019-0566-y)

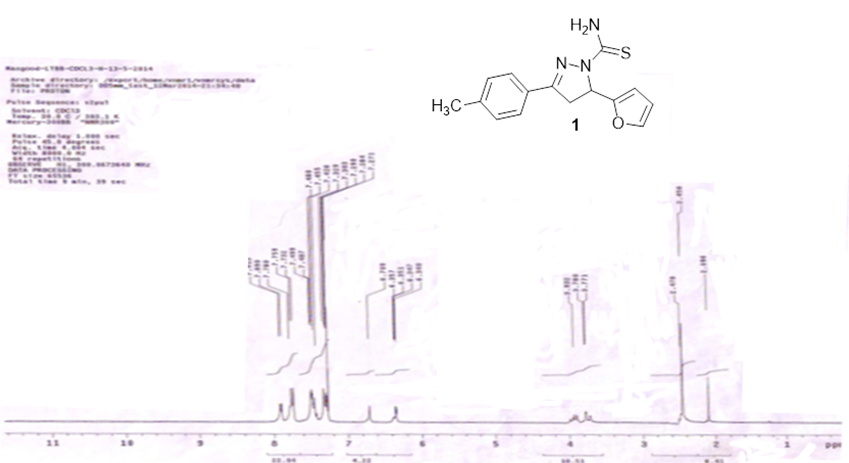


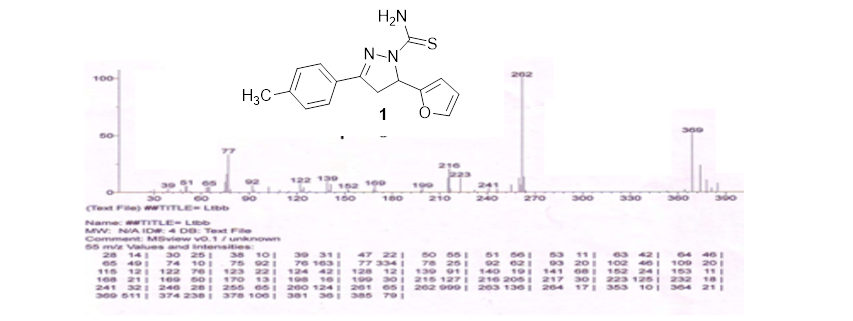


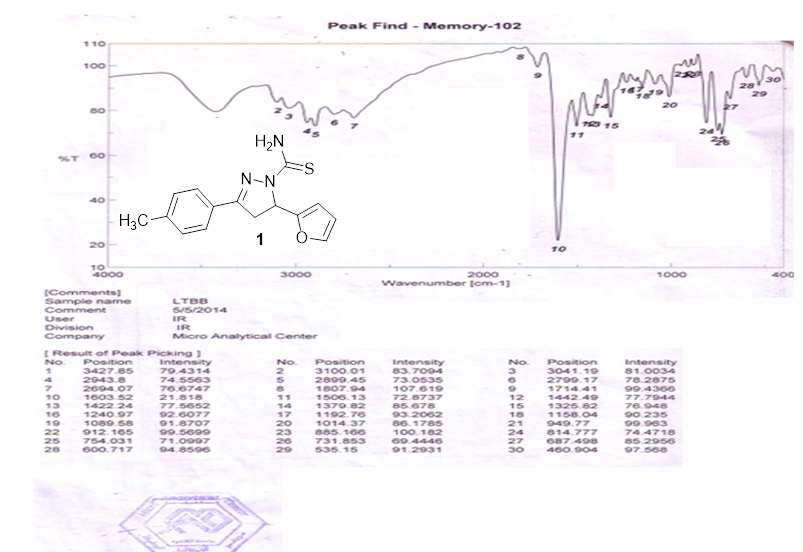


Figure S1. 1H NMR, Mass and IR spectra of compound (**1**).

Supplement: Supplementary file 1 — Additional file 1: Figure S1. 1H NMR, Mass and IR spectra of compound (1). [file 13065_2019_566_MOESM1_ESM.docx]

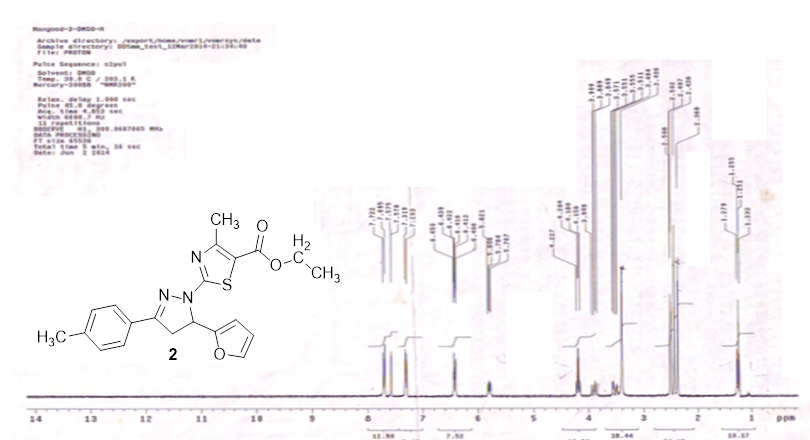


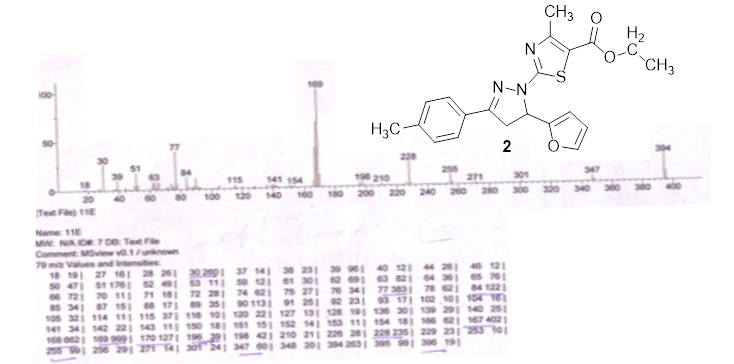


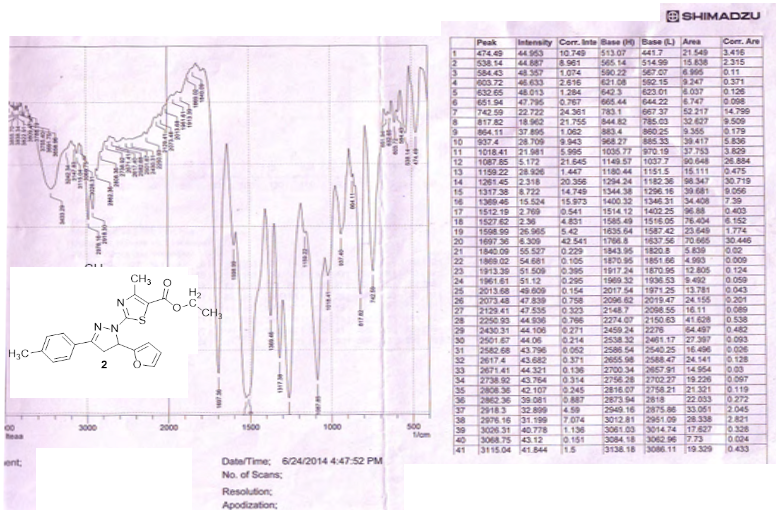


Figure S2. 1H NMR, Mass and IR spectra of compound (**2**).

Supplement: Supplementary file 2 — Additional file 2: Figure S2. 1H NMR, Mass and IR spectra of compound (2). [file 13065_2019_566_MOESM2_ESM.docx]

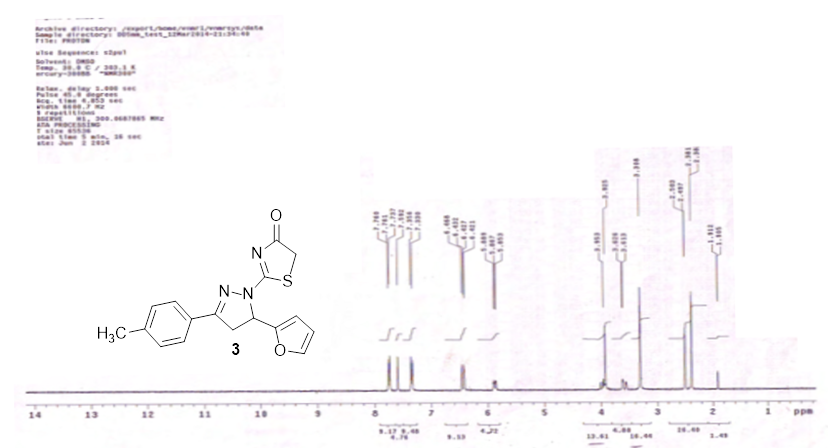


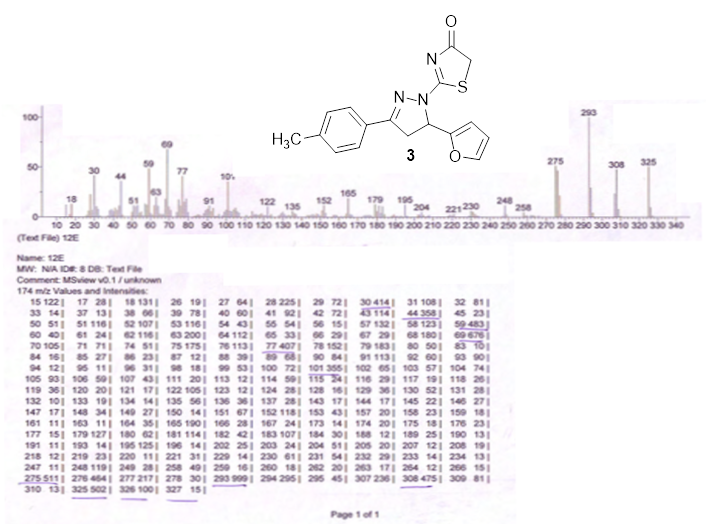


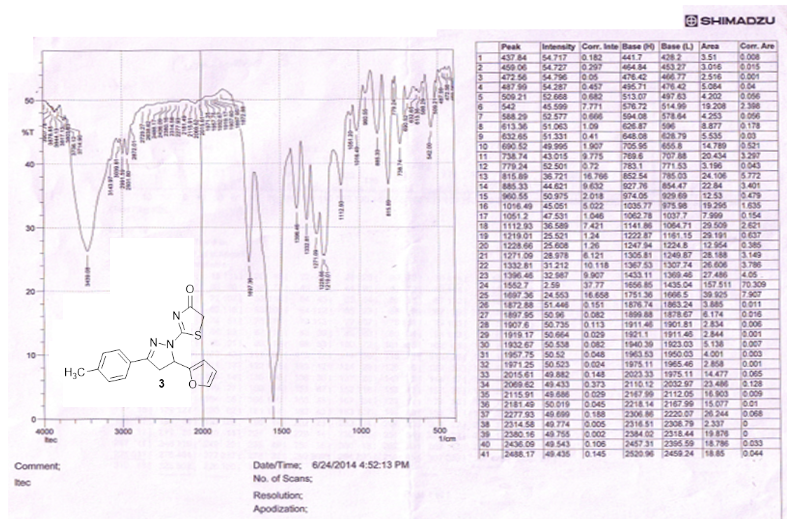


Figure S3. 1H NMR, Mass and IR spectra of compound (**3**).

Supplement: Supplementary file 3 — Additional file 3: Figure S3. 1H NMR, Mass and IR spectra of compound (3). [file 13065_2019_566_MOESM3_ESM.docx]

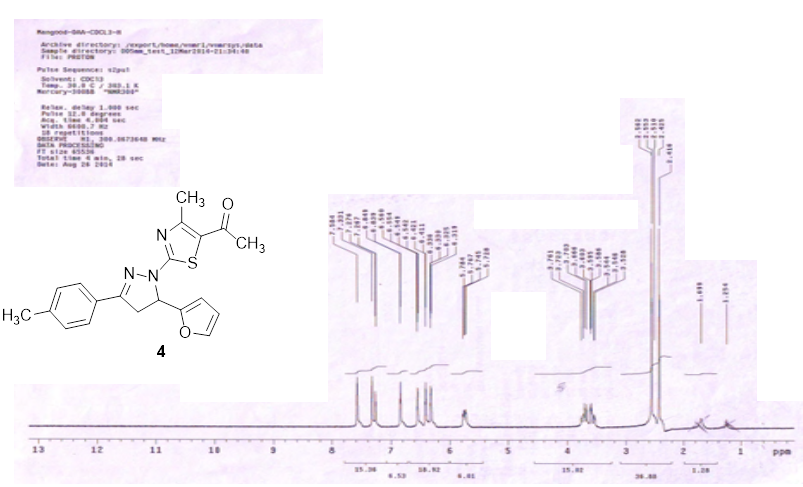


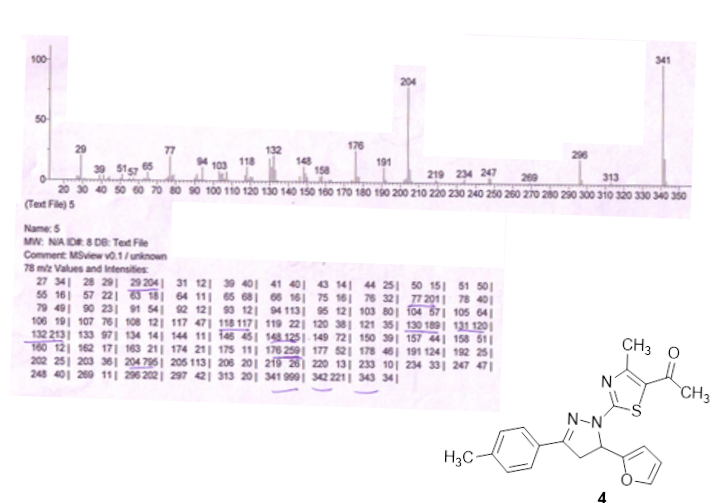


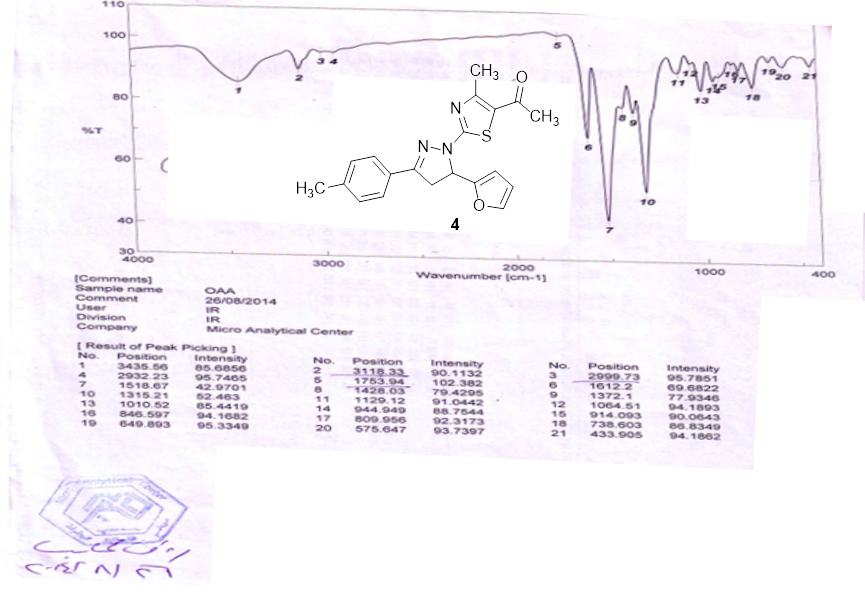


Figure S4. 1H NMR, Mass and IR spectra of compound (**4**).

Supplement: Supplementary file 4 — Additional file 4: Figure S4. 1H NMR, Mass and IR spectra of compound (4). [file 13065_2019_566_MOESM4_ESM.docx]

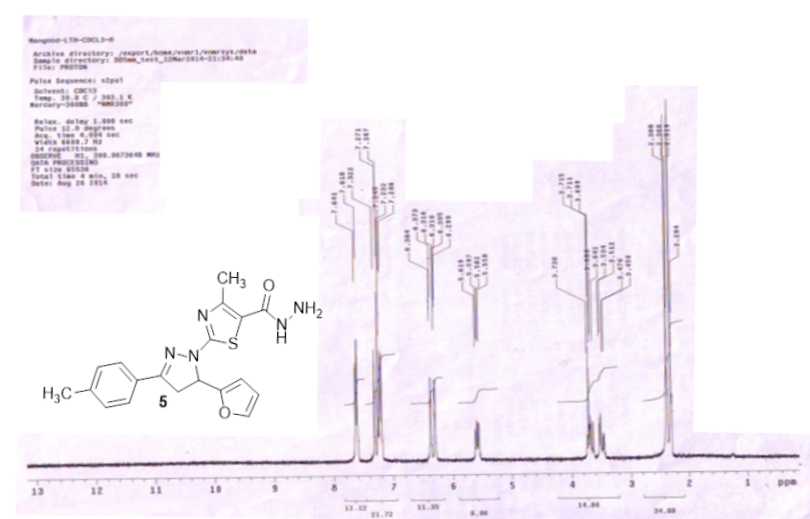


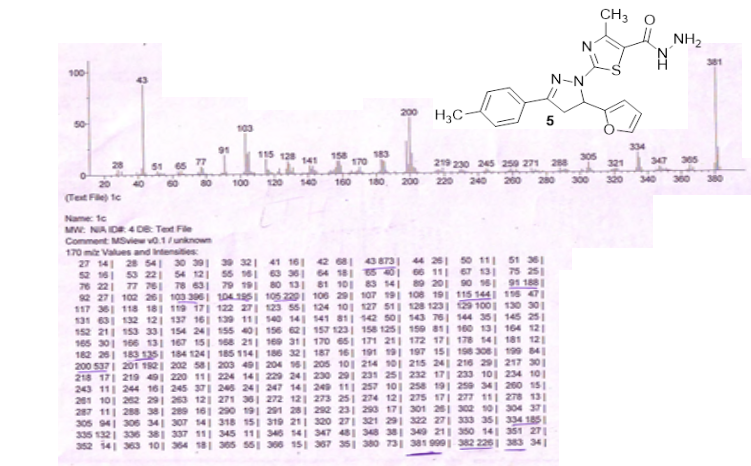


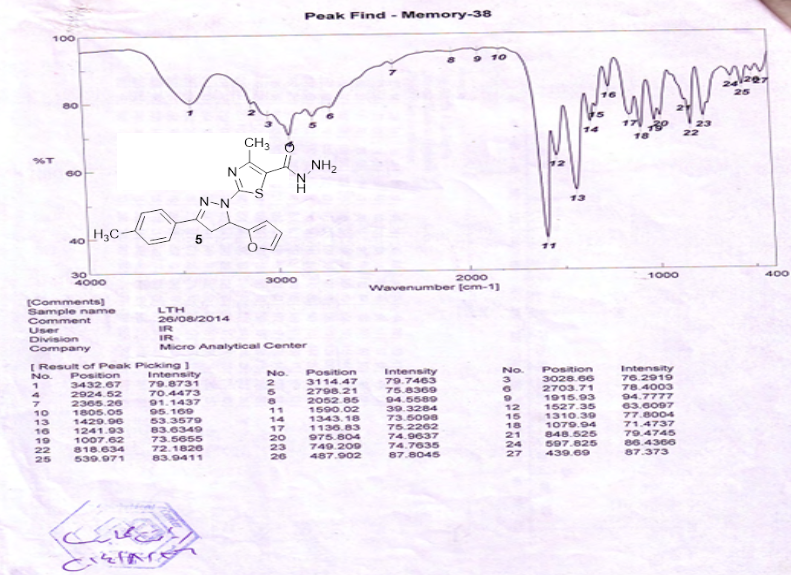


Figure S5. 1H NMR, Mass and IR spectra of compound (**5**).

Supplement: Supplementary file 5 — Additional file 5: Figure S5. 1H NMR, Mass and IR spectra of compound (5). [file 13065_2019_566_MOESM5_ESM.docx]

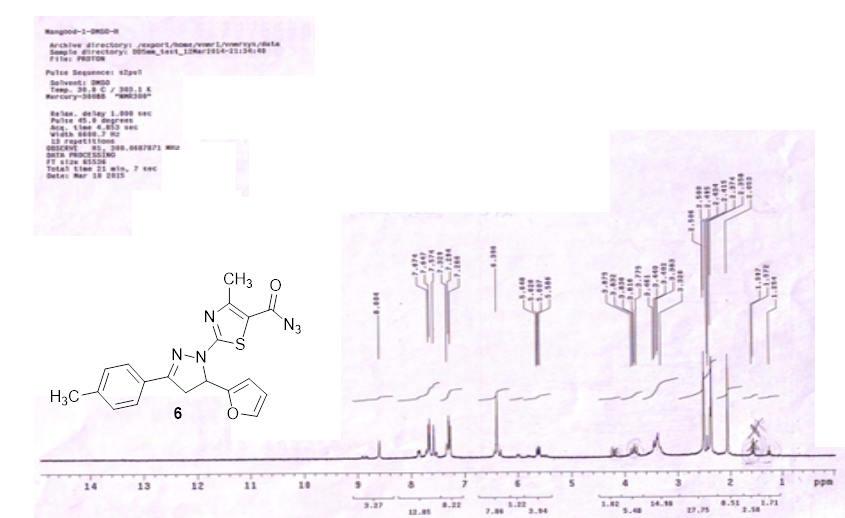


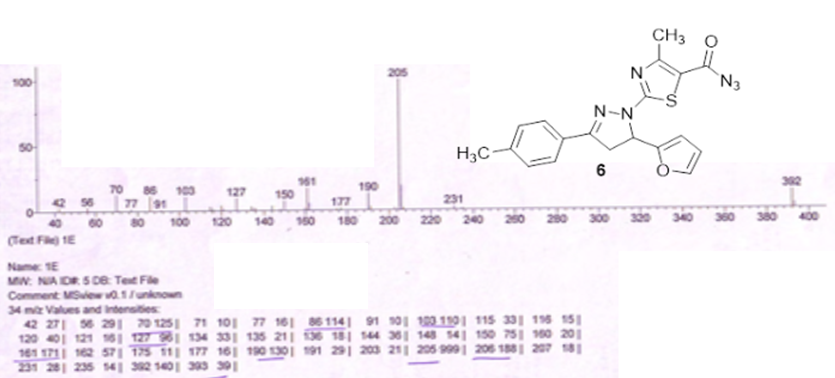


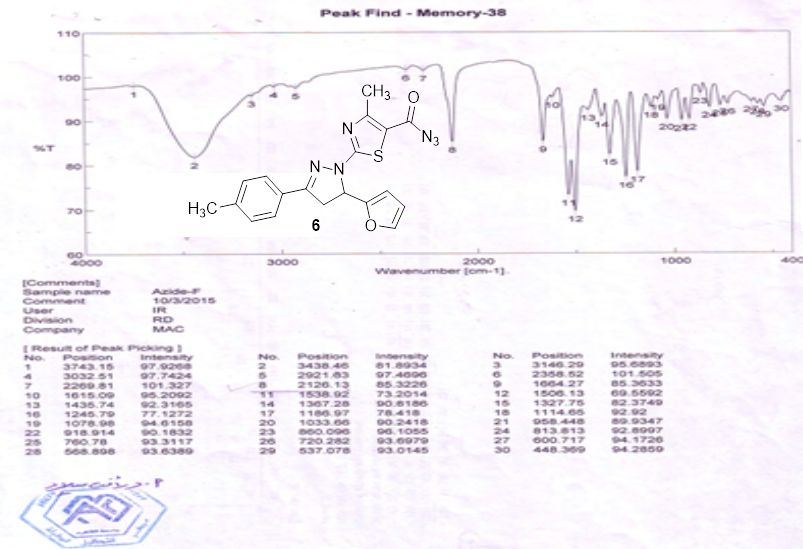


Figure S6. 1H NMR, Mass and IR spectra of compound (**6**).

Supplement: Supplementary file 6 — Additional file 6: Figure S6. 1H NMR, Mass and IR spectra of compound (6). [file 13065_2019_566_MOESM6_ESM.docx]

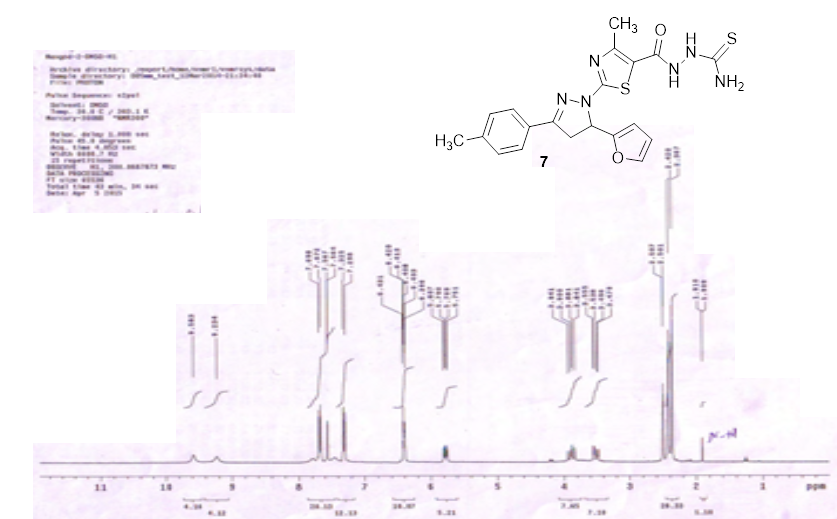


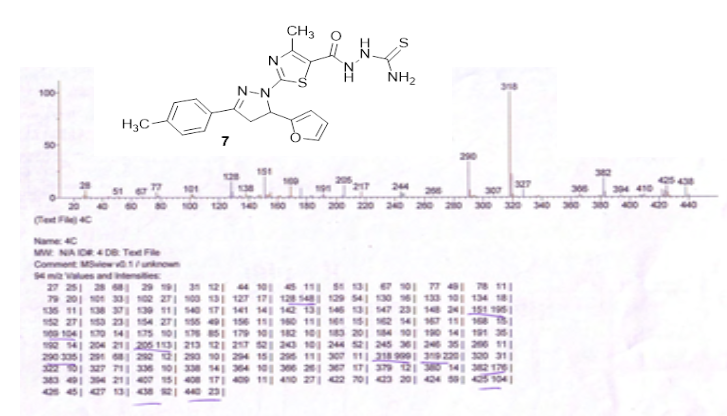


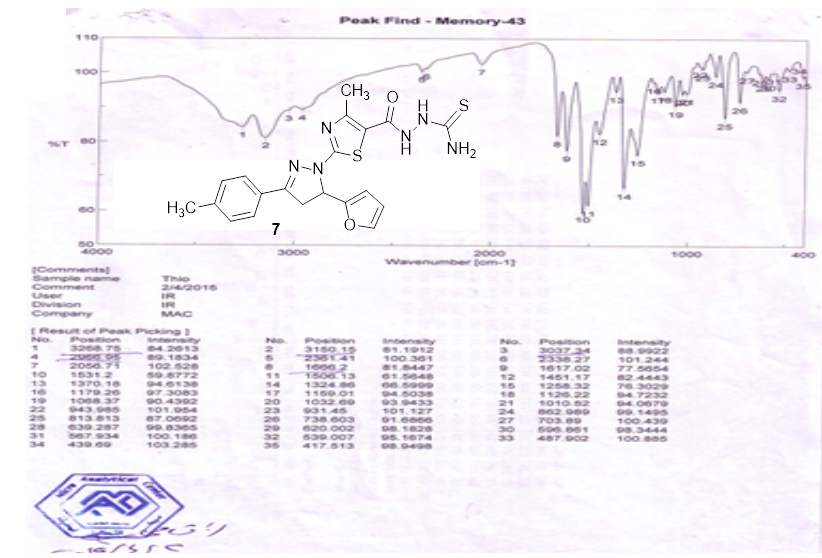


Figure S7. 1H NMR, Mass and IR spectra of compound (**7**).

Supplement: Supplementary file 7 — Additional file 7: Figure S7. 1H NMR, Mass and IR spectra of compound (7). [file 13065_2019_566_MOESM7_ESM.docx]

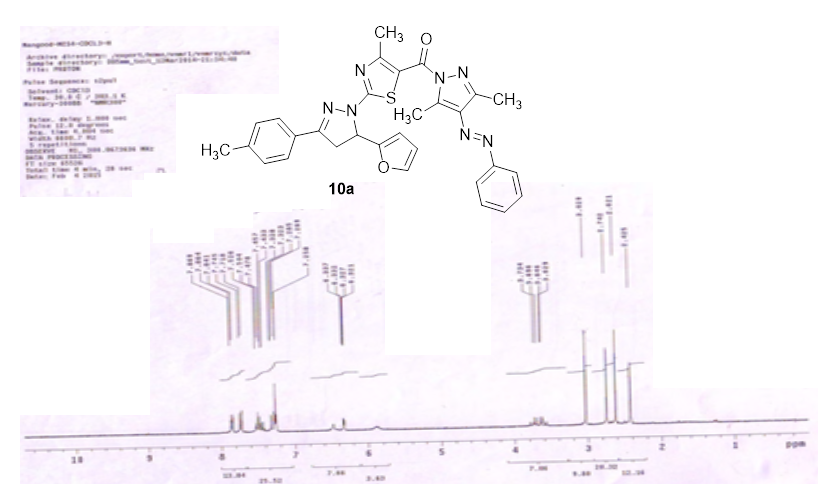


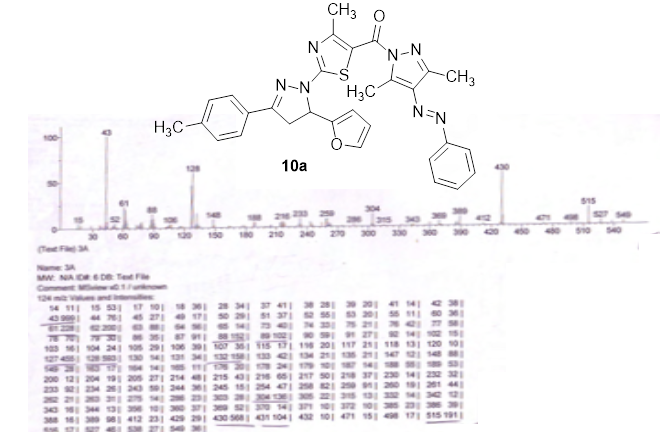


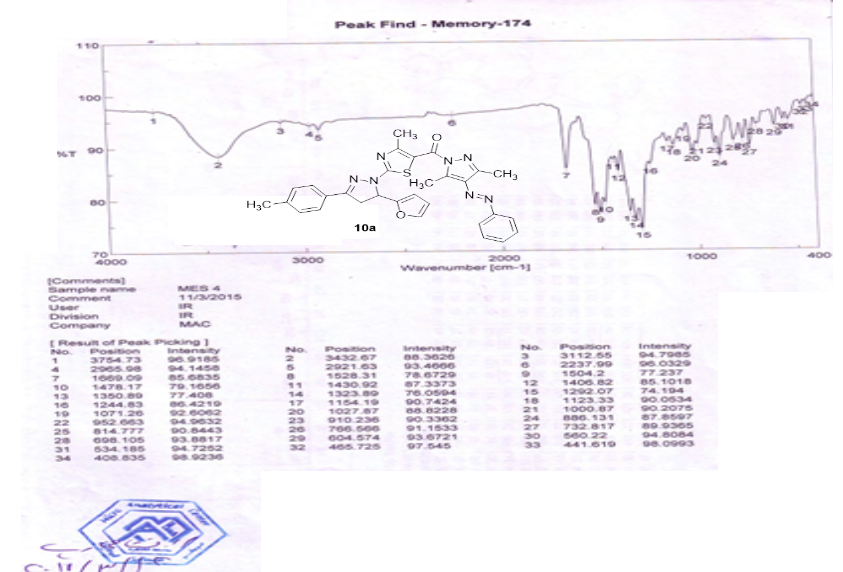


Figure S8. 1H NMR, Mass, and IR spectra of compound (**10a**).

Supplement: Supplementary file 8 — Additional file 8: Figure S8. 1H NMR, Mass, and IR spectra of compound (10a). [file 13065_2019_566_MOESM8_ESM.docx]

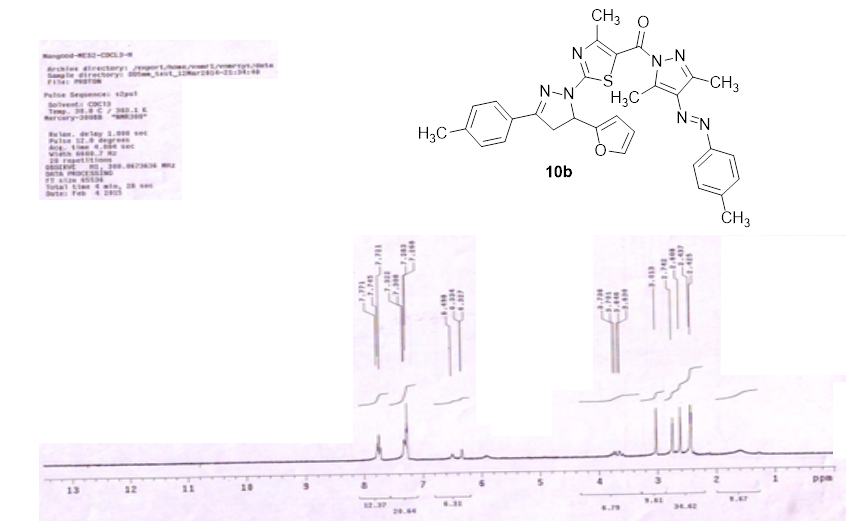


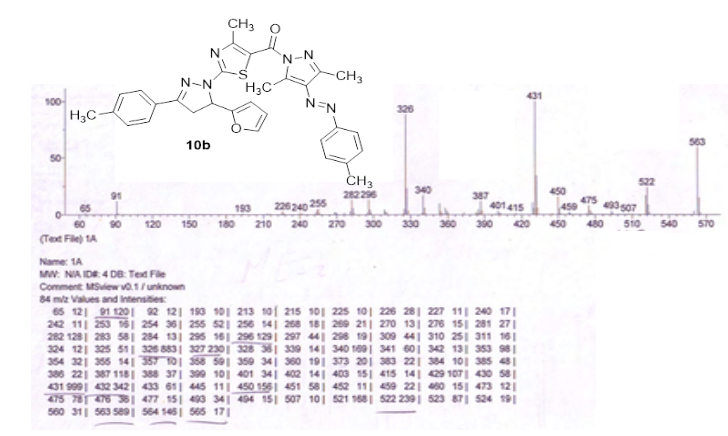


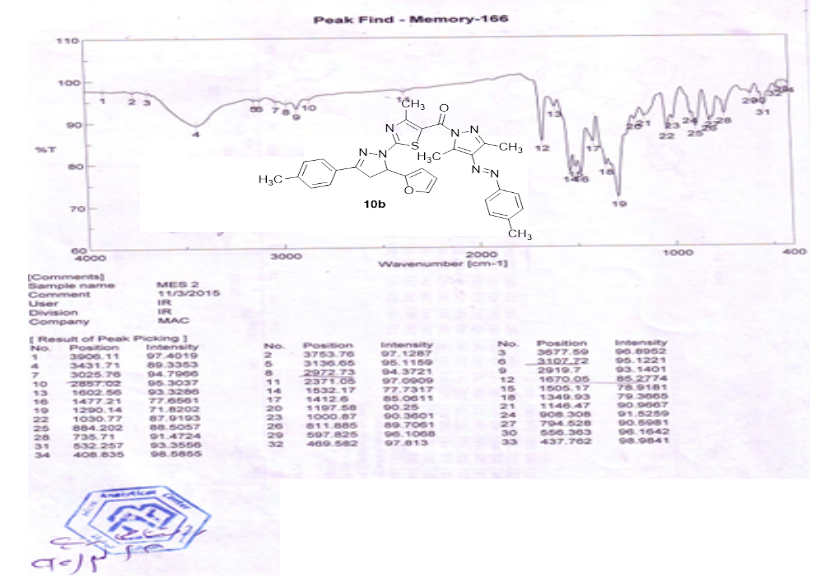


Figure S9. 1H NMR, Mass and IR spectra of compound (**10b**).

Supplement: Supplementary file 9 — Additional file 9: Figure S9. 1H NMR, Mass and IR spectra of compound (10b). [file 13065_2019_566_MOESM9_ESM.docx]

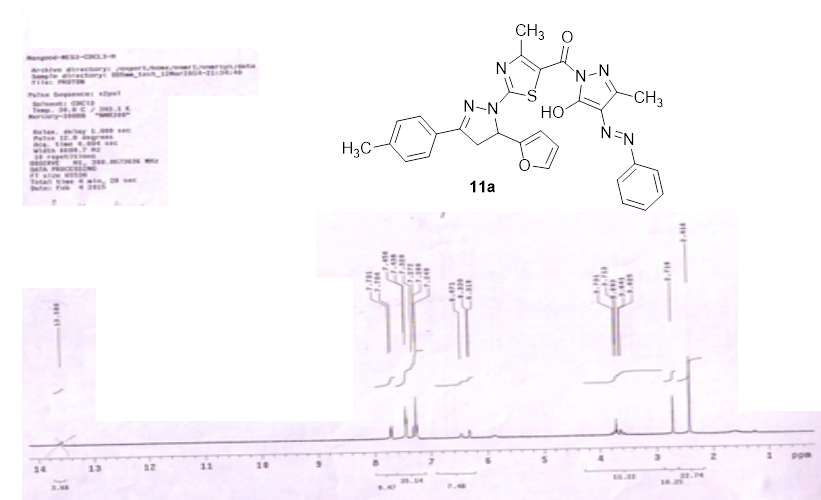


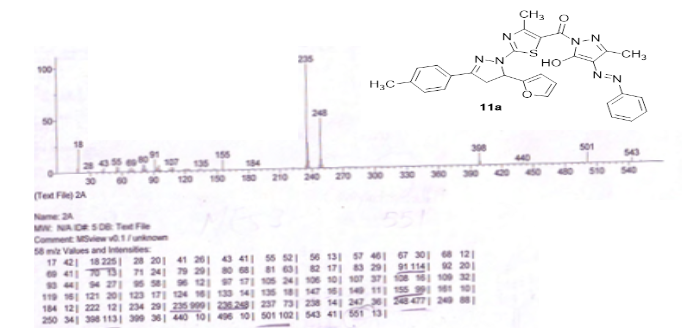


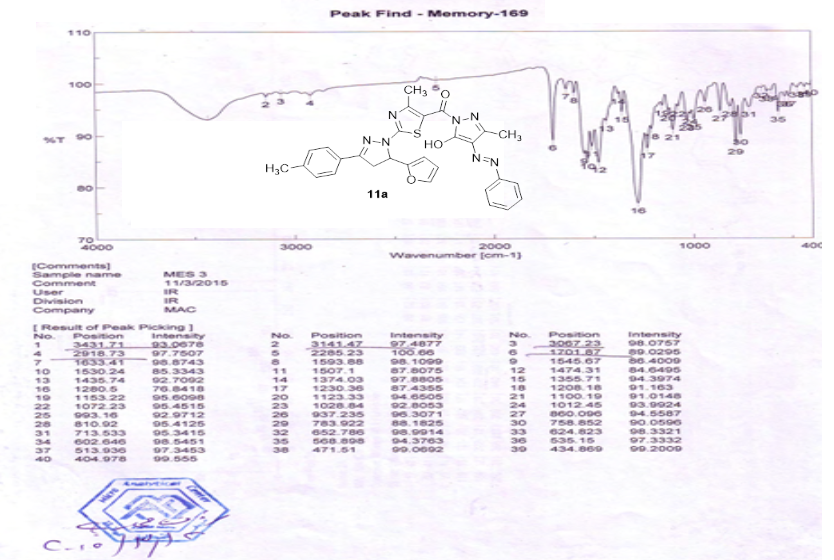


Figure S10. 1H NMR, Mass and IR spectra of compound (**11a**).

Supplement: Supplementary file 10 — Additional file 10: Figure S10. 1H NMR, Mass and IR spectra of compound (11a). [file 13065_2019_566_MOESM10_ESM.docx]

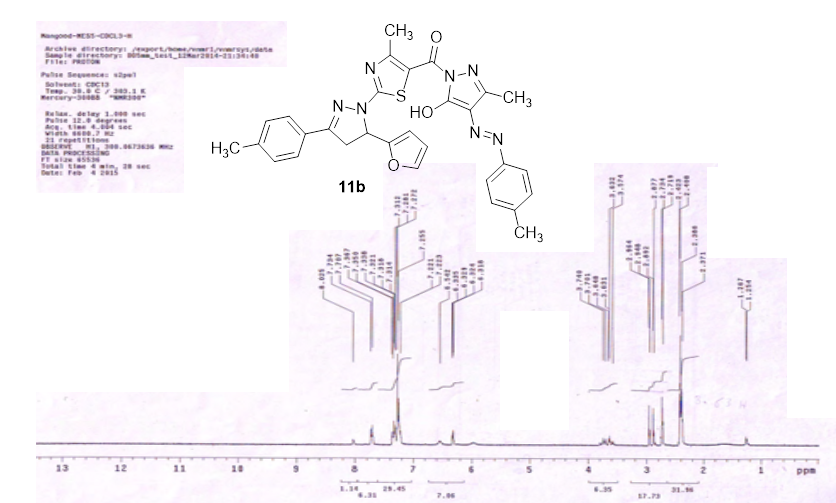


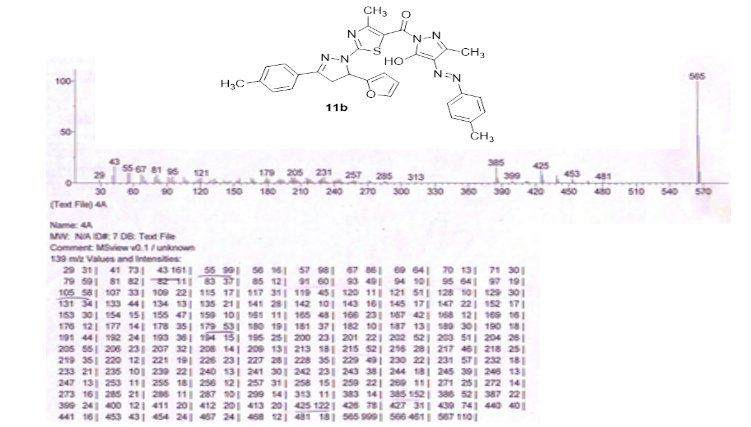


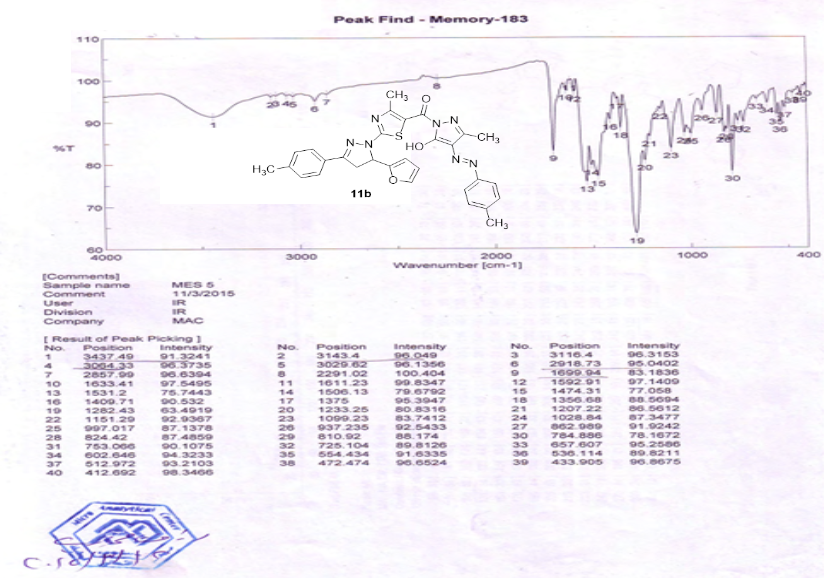


Figure S11. 1H NMR, Mass, and IR spectra of compound (**11b**).

Supplement: Supplementary file 11 — Additional file 11: Figure S11. 1H NMR, Mass, and IR spectra of compound (11b). [file 13065_2019_566_MOESM11_ESM.docx]

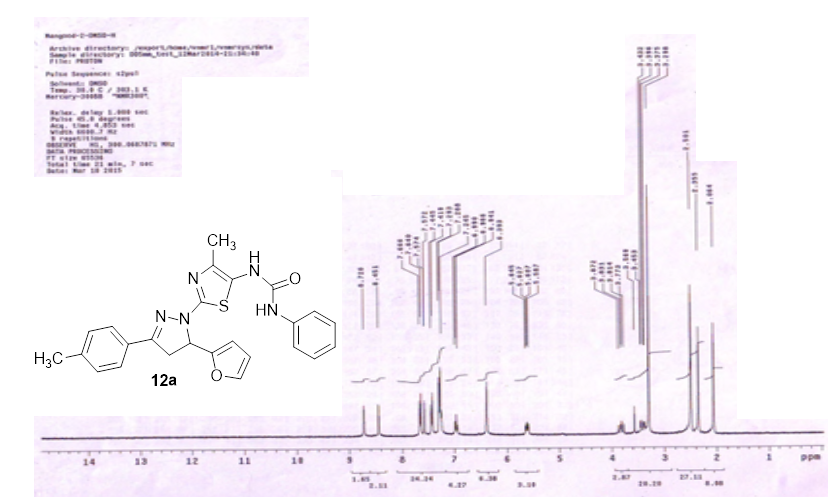


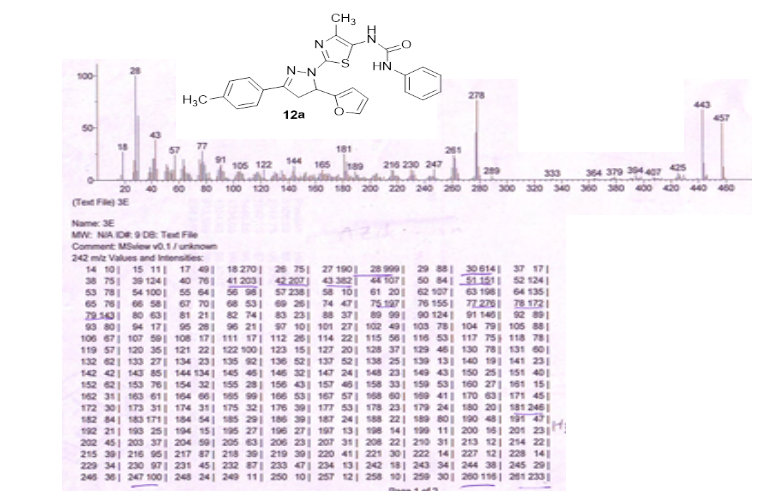


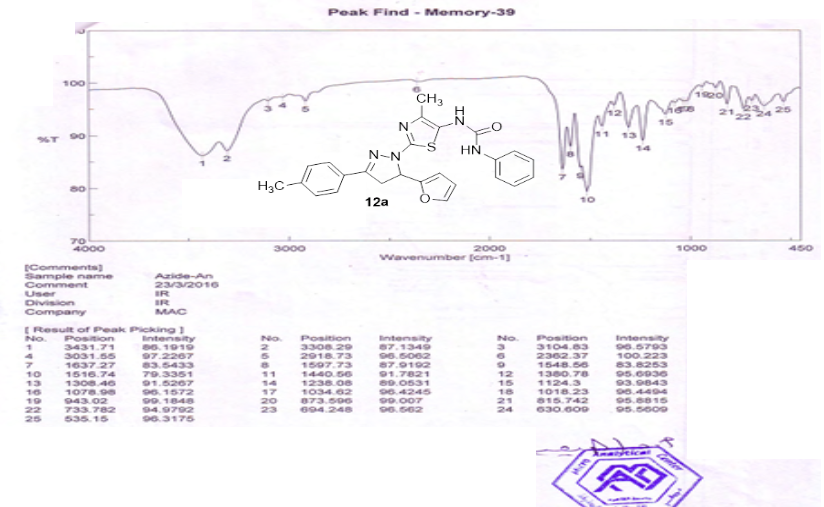


Figure S12. 1H NMR, Mass and IR spectra of compound (**12a**).

Supplement: Supplementary file 12 — Additional file 12: Figure S12. 1H NMR, Mass and IR spectra of compound (12a). [file 13065_2019_566_MOESM12_ESM.docx]

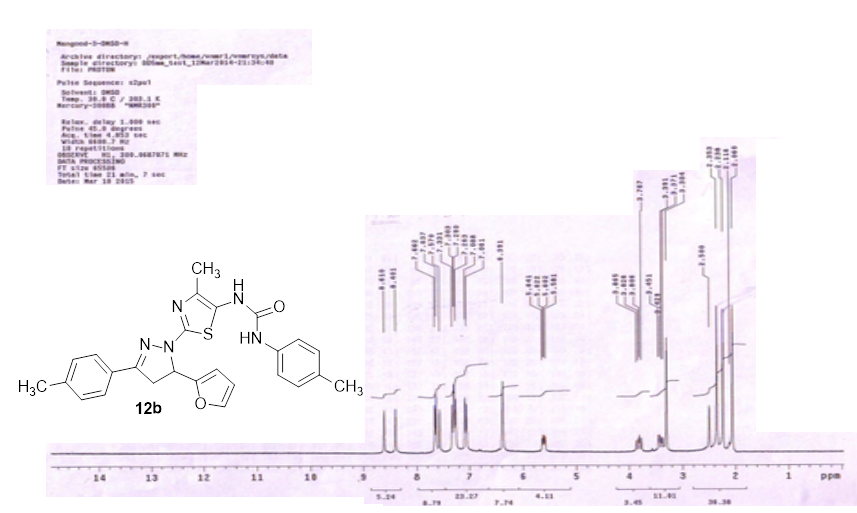


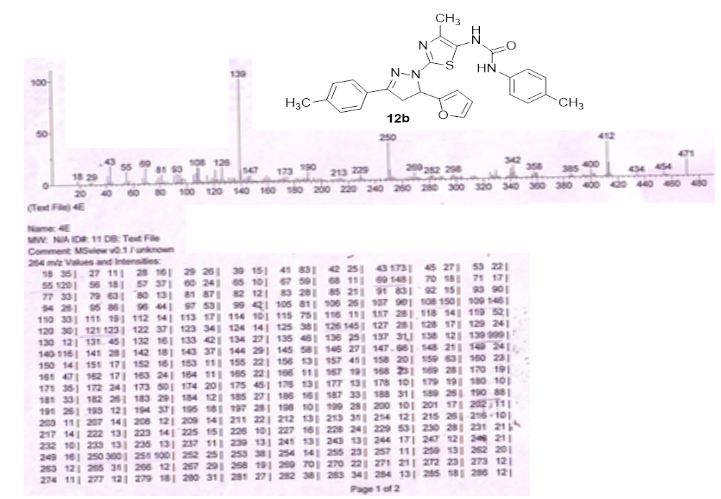


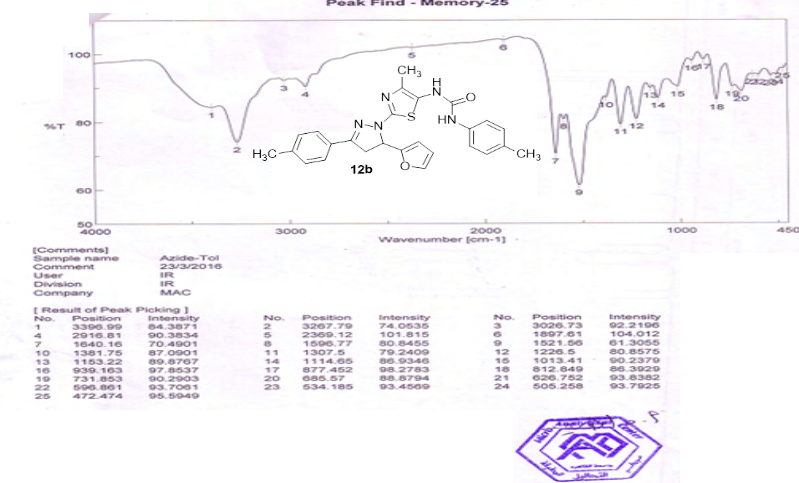


Figure S13. 1H NMR, Mass and IR spectra of compound (**12b**).

Supplement: Supplementary file 13 — Additional file 13: Figure S13. 1H NMR, Mass and IR spectra of compound (12b). [file 13065_2019_566_MOESM13_ESM.docx]

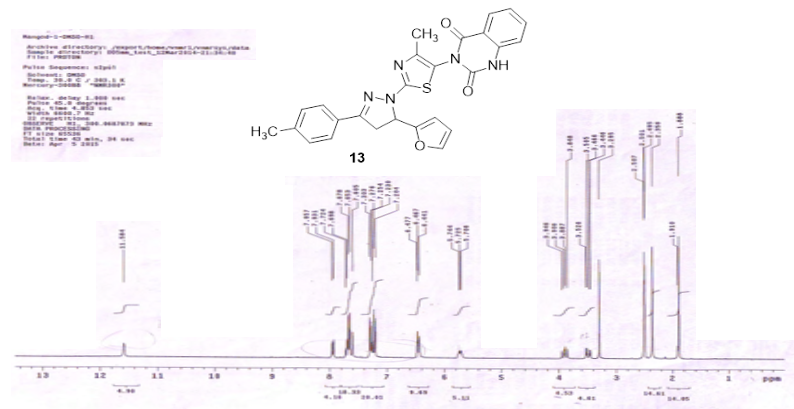


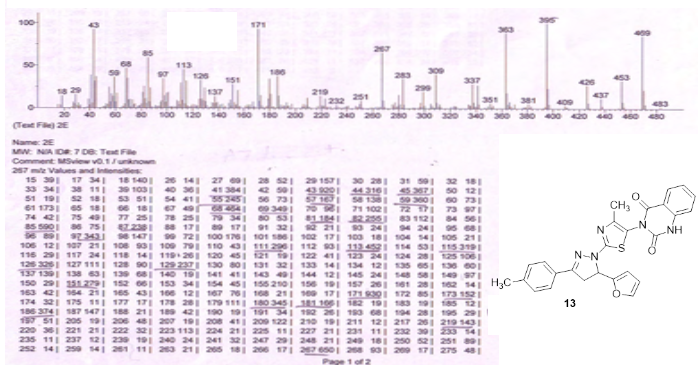


Figure S14. 1H NMR, and Mass spectra of compound (**13**).

Supplement: Supplementary file 14 — Additional file 14: Figure S14. 1H NMR and Mass spectra of compound (13). [file 13065_2019_566_MOESM14_ESM.docx]

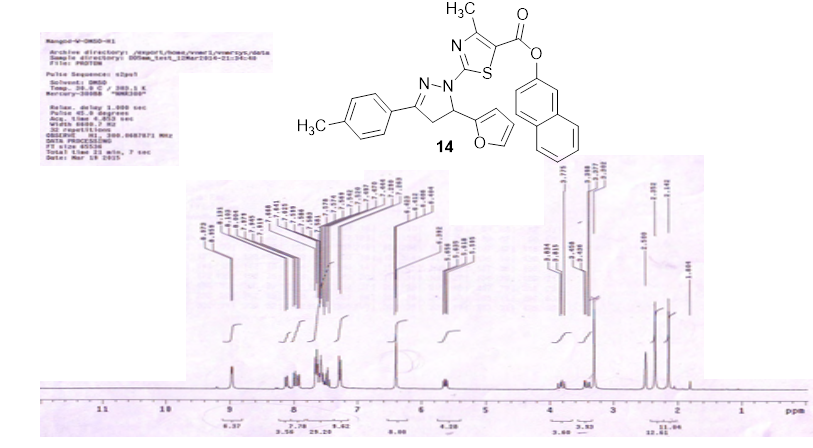


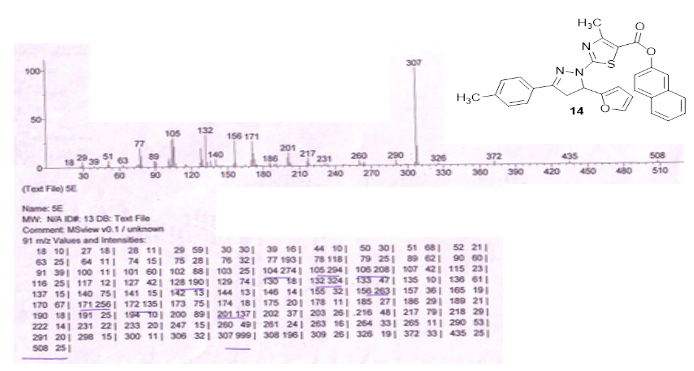


Figure S15. 1H NMR and Mass spectra of compound (**14**).

Supplement: Supplementary file 15 — Additional file 15: Figure S15. 1H NMR and Mass spectra of compound (14). [file 13065_2019_566_MOESM15_ESM.docx]

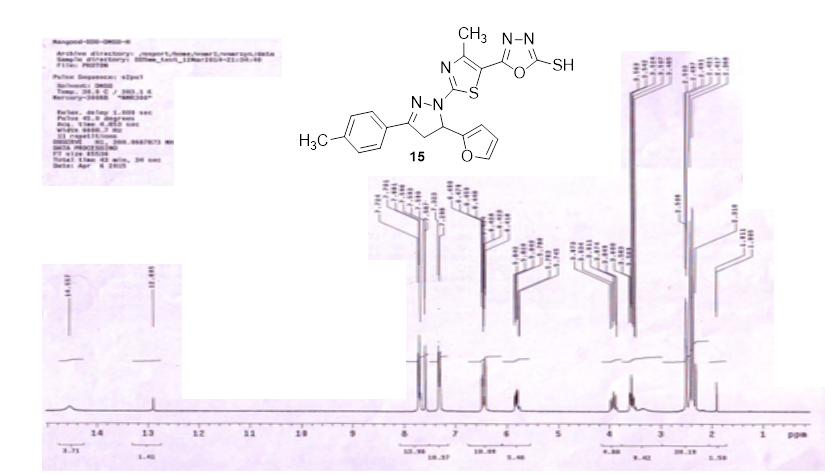


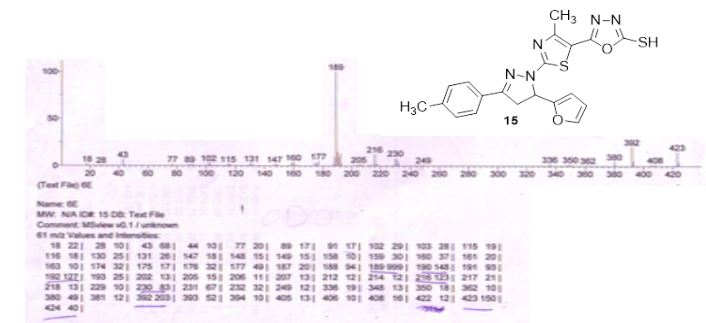


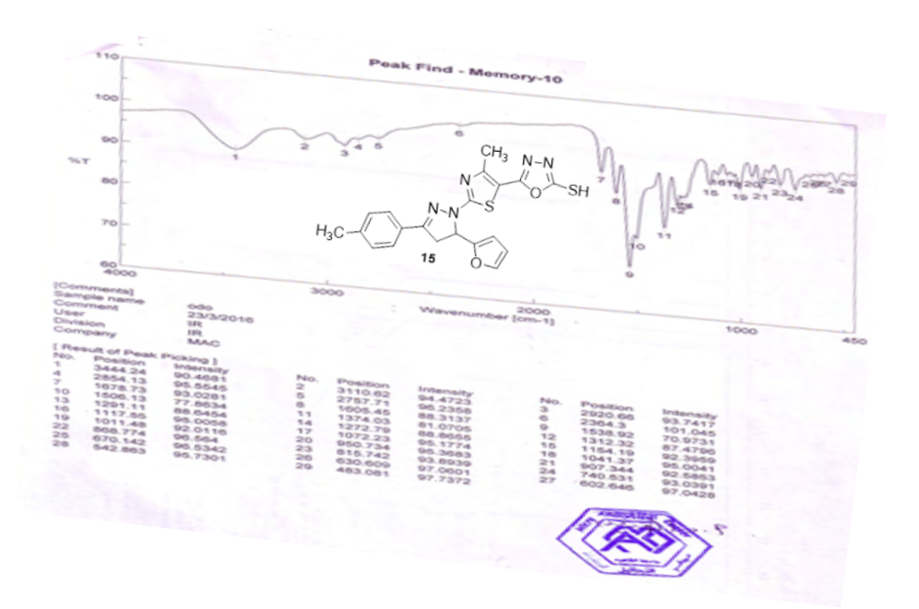


Figure S16. 1H NMR, Mass and IR spectra of compound (**15**).

Supplement: Supplementary file 16 — Additional file 16: Figure S16. 1H NMR, Mass and IR spectra of compound (15). [file 13065_2019_566_MOESM16_ESM.docx]

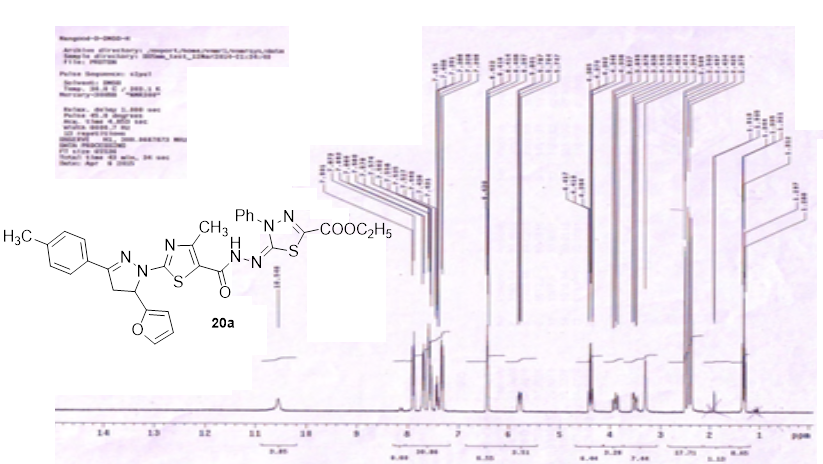


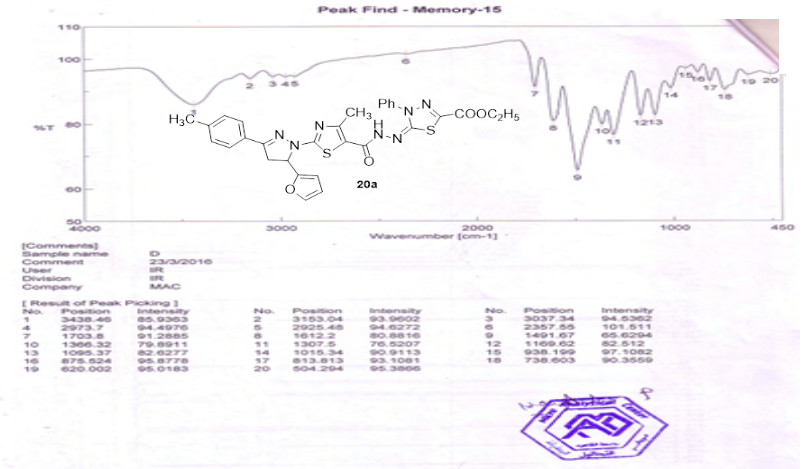


Figure S17. 1H NMR and IR spectra of compound (**20a**).

Supplement: Supplementary file 17 — Additional file 17: Figure S17. 1H NMR and IR spectra of compound (20a). [file 13065_2019_566_MOESM17_ESM.docx]

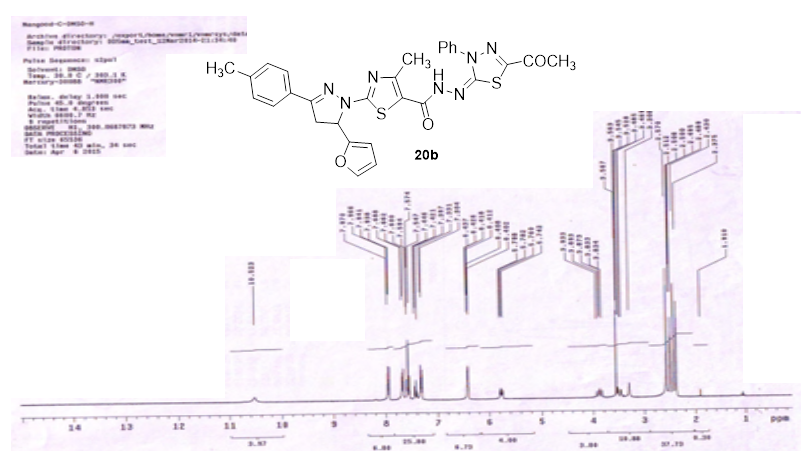


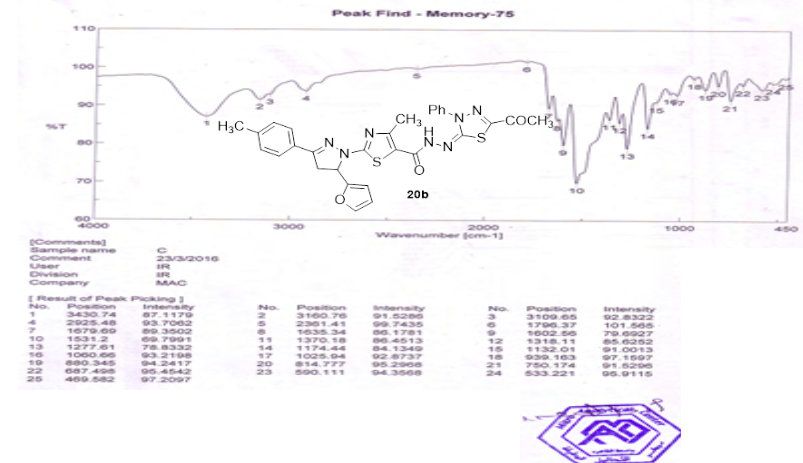


Figure S18. 1H NMR and IR spectra of compound (**20b**).

Supplement: Supplementary file 18 — Additional file 18: Figure S18. 1H NMR and IR spectra of compound (20b). [file 13065_2019_566_MOESM18_ESM.docx]

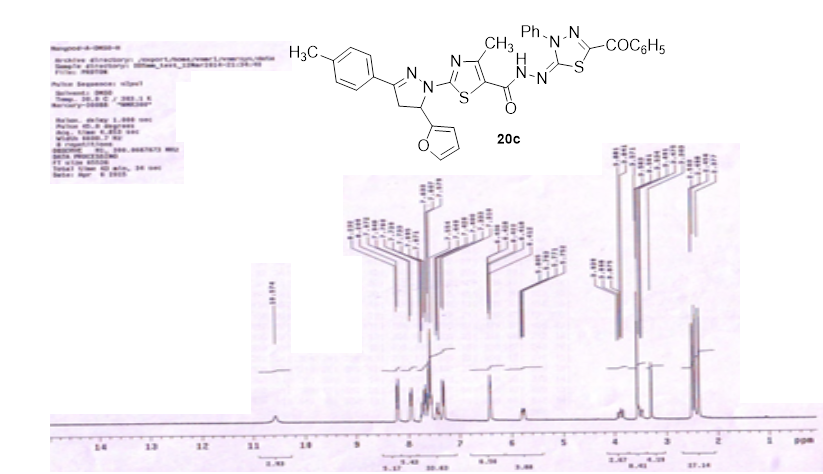


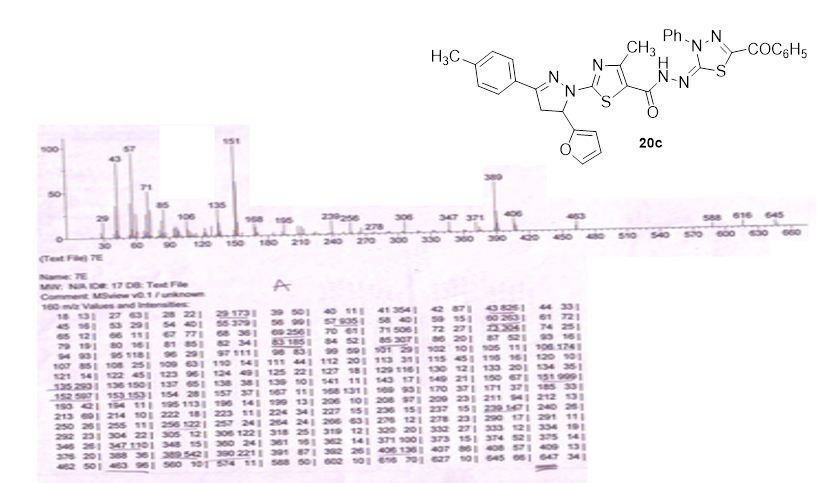


Figure S19. 1H NMR and Mass spectra of compound (**20c**).

Supplement: Supplementary file 19 — Additional file 19: Figure S19. 1H NMR and Mass spectra of compound (20c). [file 13065_2019_566_MOESM19_ESM.docx]

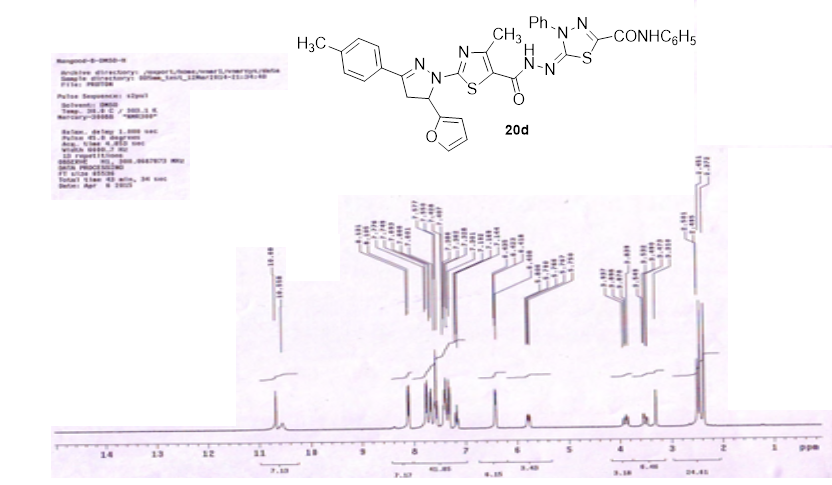


Figure S20. 1H NMR spectra of compound (**20d**).

Supplement: Supplementary file 20 — Additional file 20: Figure S20. 1H NMR spectra of compound (20d). [file 13065_2019_566_MOESM20_ESM.docx]

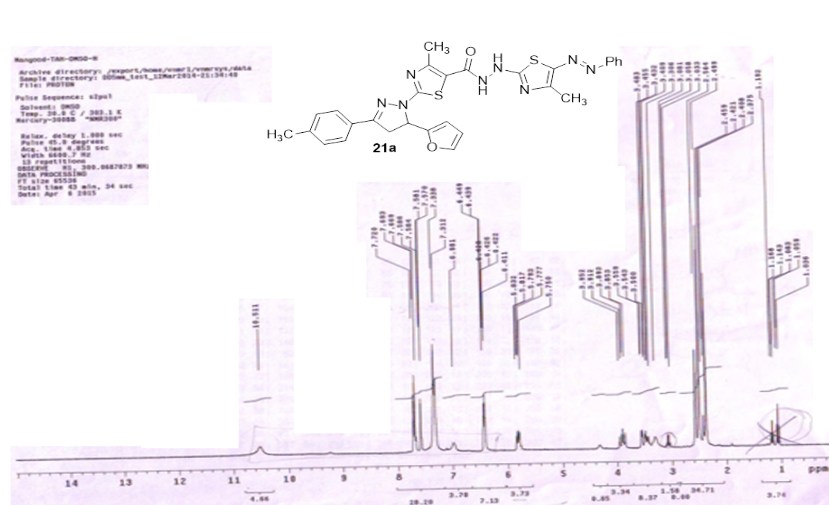


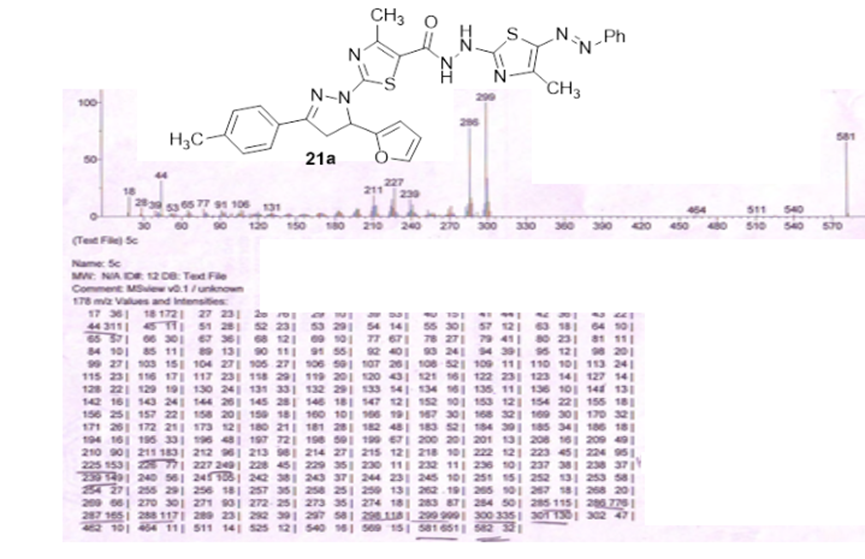


Figure S21. 1H NMR and Mass spectra of compound (**21a**).

Supplement: Supplementary file 21 — Additional file 21: Figure S21. 1H NMR and Mass spectra of compound (21a). [file 13065_2019_566_MOESM21_ESM.docx]

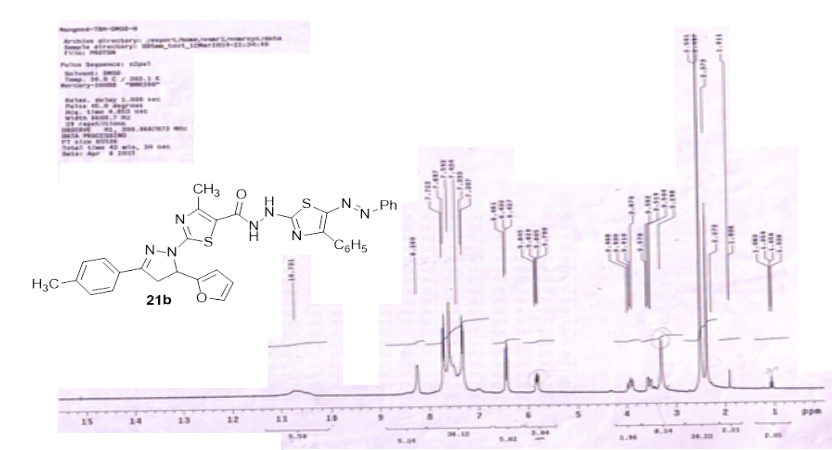


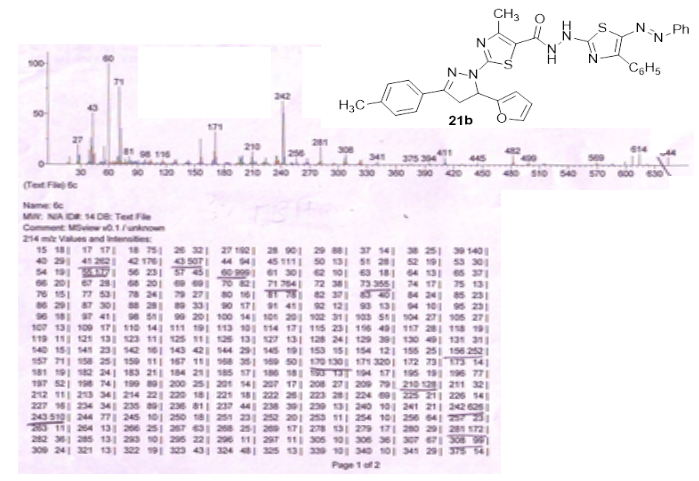


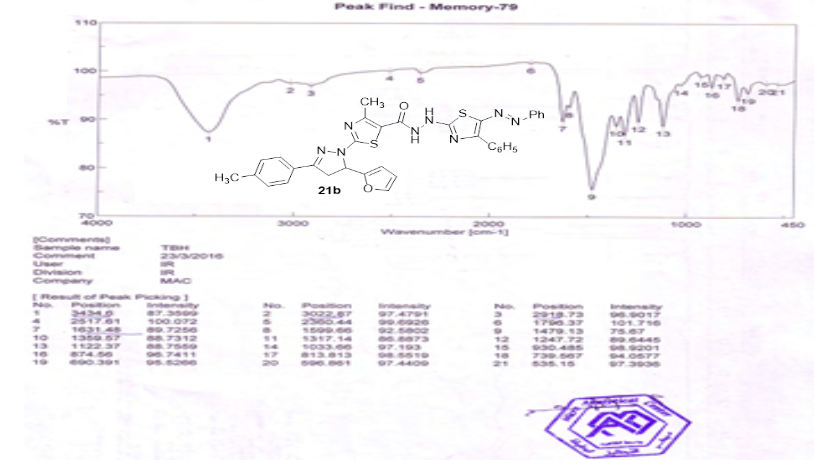


Figure S22. 1H NMR, Mass and IR spectra of compound (**21b**).

Supplement: Supplementary file 22 — Additional file 22: Figure S22. 1H NMR, Mass and IR spectra of compound (21b). [file 13065_2019_566_MOESM22_ESM.docx]

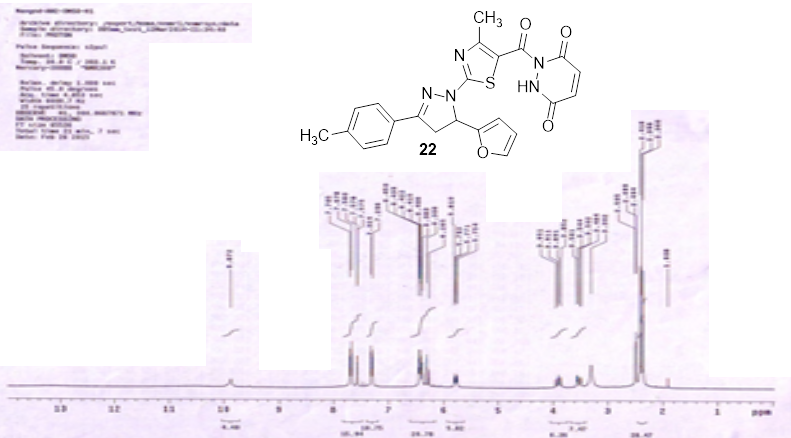


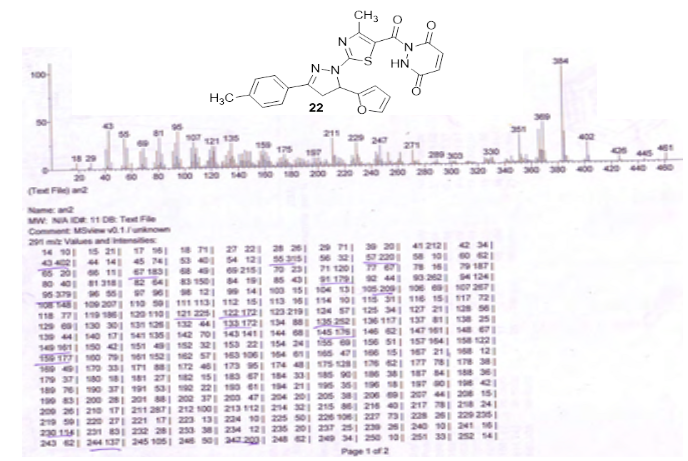


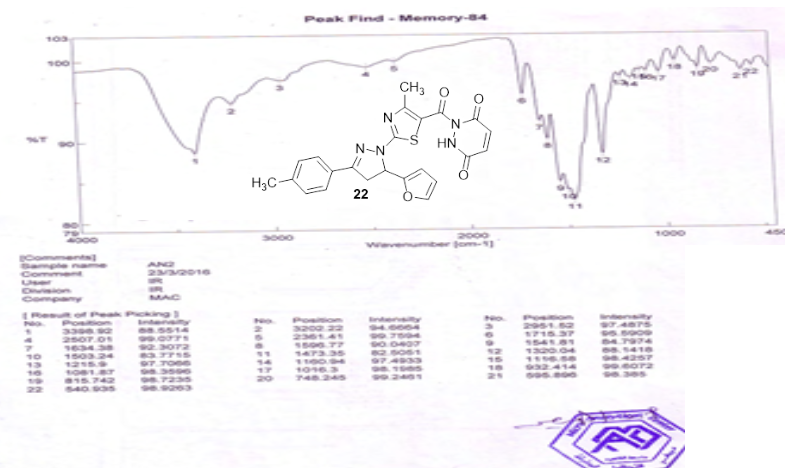


Figure S23. 1H NMR, Mass and IR spectra of compound (**22**).

Supplement: Supplementary file 23 — Additional file 23: Figure S23. 1H NMR, Mass and IR spectra of compound (22). [file 13065_2019_566_MOESM23_ESM.docx]

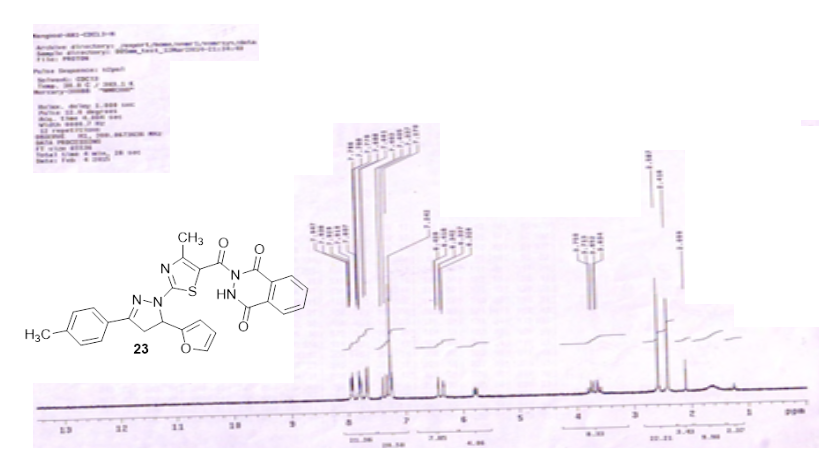


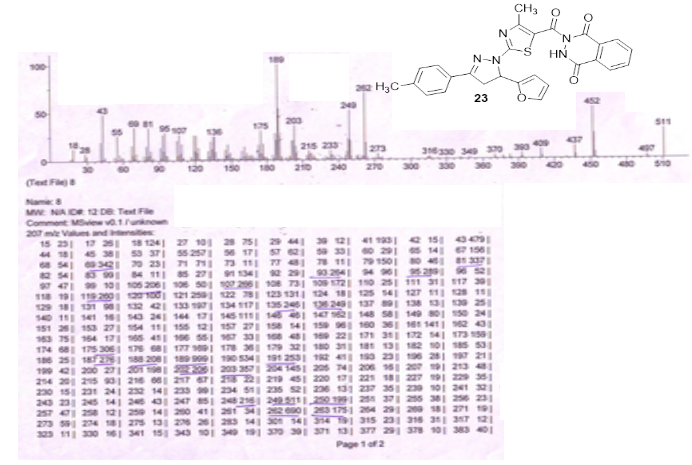


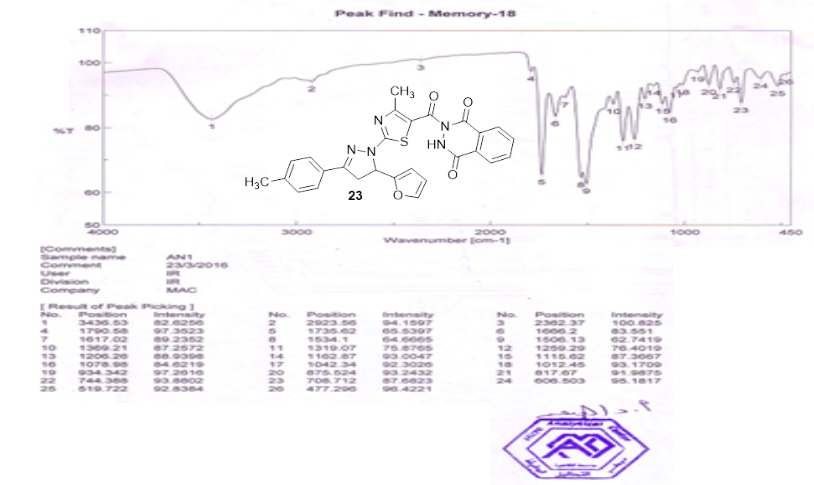


Figure S24. 1H NMR, Mass and IR spectra of compound (**23**).

Supplement: Supplementary file 24 — Additional file 24: Figure S24. 1H NMR, Mass and IR spectra of compound (23). [file 13065_2019_566_MOESM24_ESM.docx]

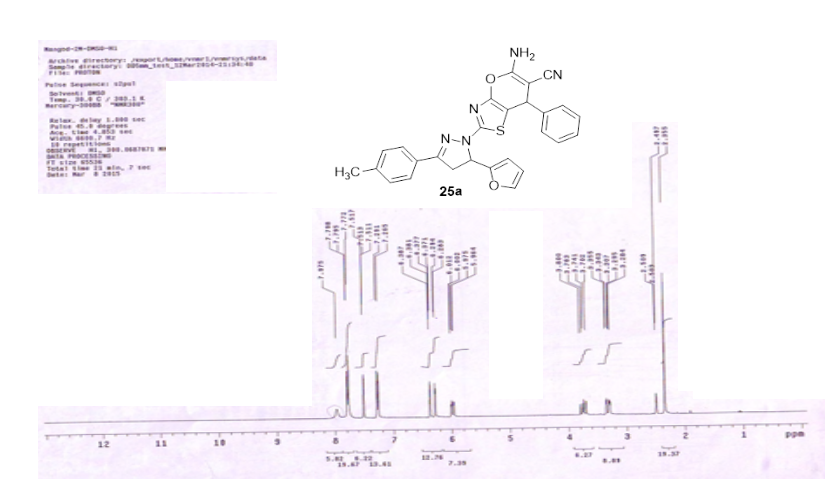


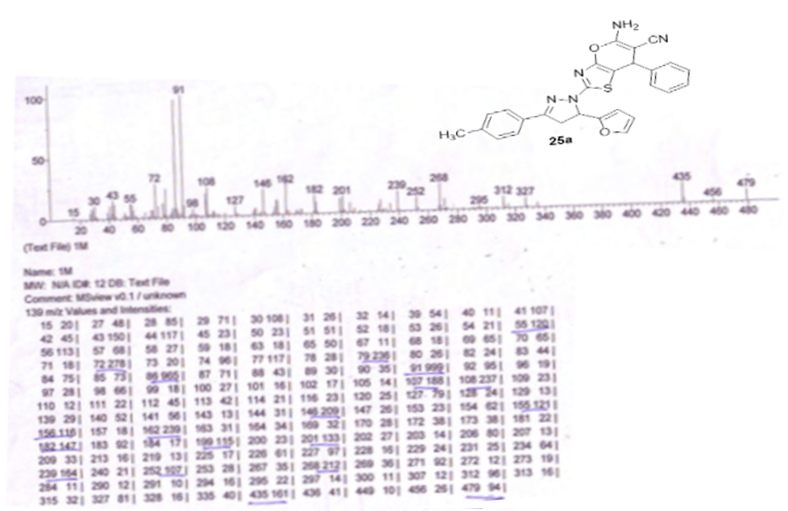


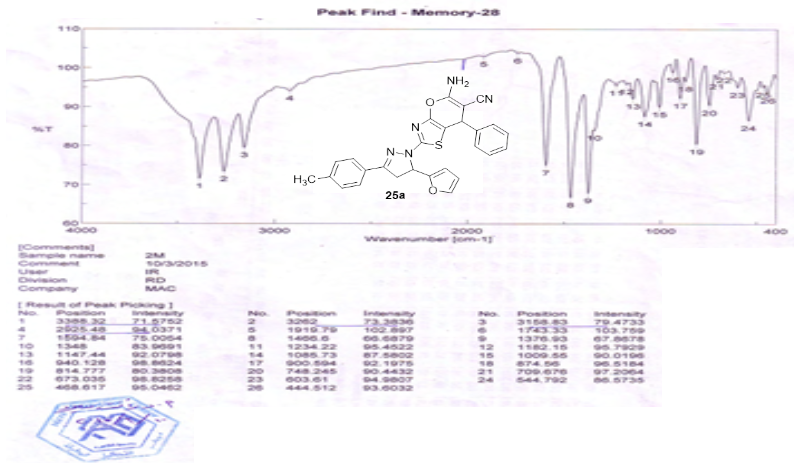


Figure S25. 1H NMR, Mass and IR spectra of compound (**25a**).

Supplement: Supplementary file 25 — Additional file 25: Figure S25. 1H NMR, Mass and IR spectra of compound (25a). [file 13065_2019_566_MOESM25_ESM.docx]

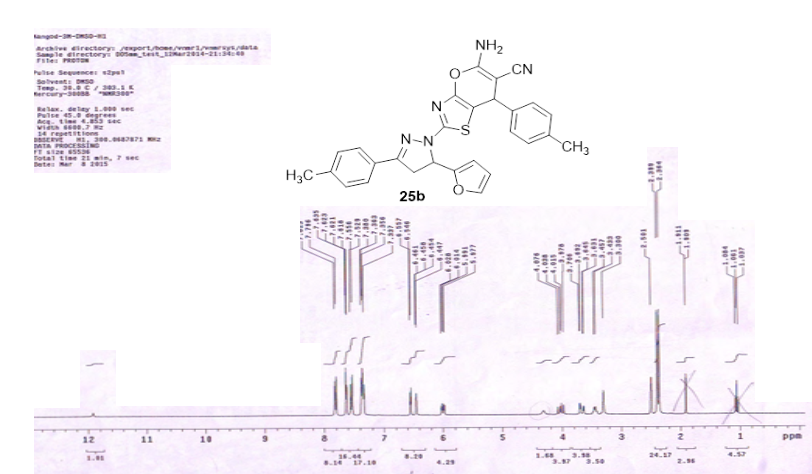


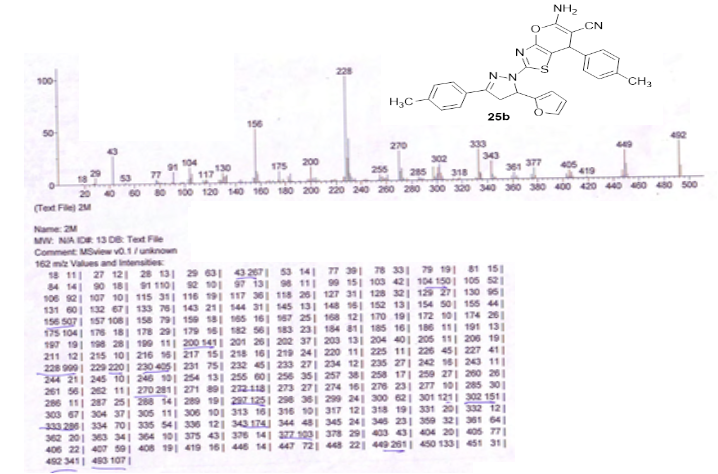


Figure S26. 1H NMR and Mass spectra of compound (**25b**).

Supplement: Supplementary file 26 — Additional file 26: Figure S26. 1H NMR and Mass spectra of compound (25b). [file 13065_2019_566_MOESM26_ESM.docx]

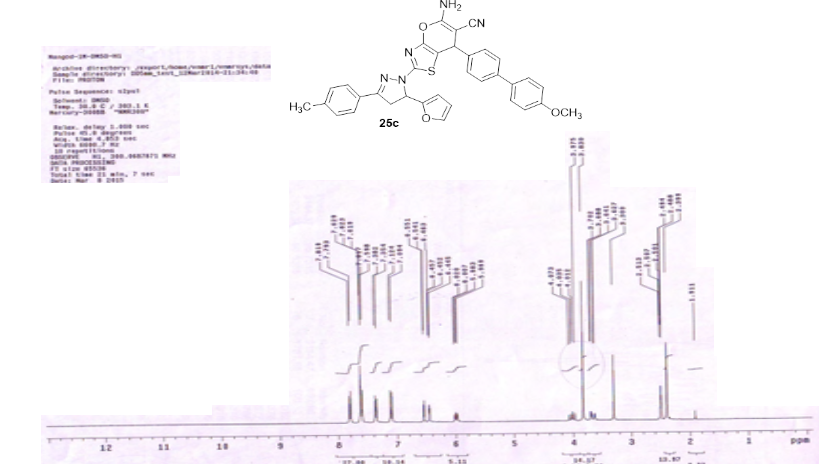


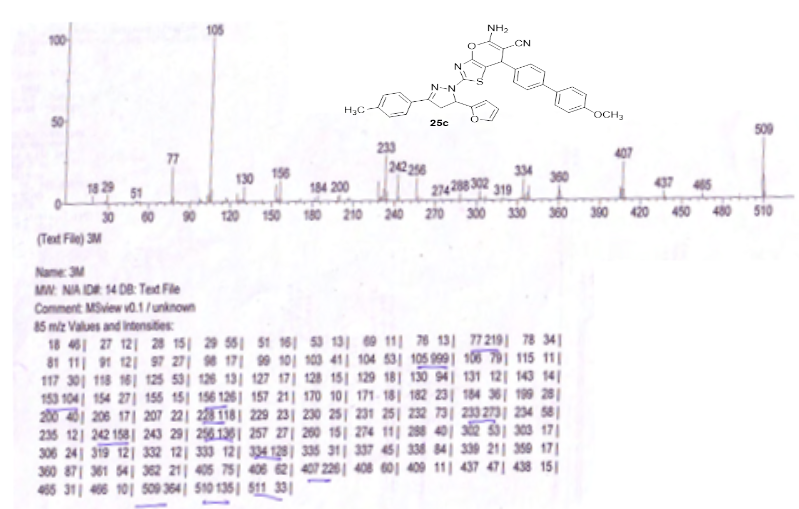


Figure S27. 1H NMR and Mass spectra of compound (**25c**).

Supplement: Supplementary file 27 — Additional file 27: Figure S27. 1H NMR and Mass spectra of compound (25c). [file 13065_2019_566_MOESM27_ESM.docx]
